# Benzohydrazide and Phenylacetamide Scaffolds: New Putative ParE Inhibitors

**DOI:** 10.3389/fbioe.2021.669728

**Published:** 2021-06-17

**Authors:** Vidyasrilekha Yele, Bharat Kumar Reddy Sanapalli, Ashish D. Wadhwani, Afzal Azam Mohammed

**Affiliations:** ^1^Department of Pharmaceutical Chemistry, JSS College of Pharmacy, JSS Academy of Higher Education & Research, Ooty, India; ^2^Department of Pharmacology, JSS College of Pharmacy, JSS Academy of Higher Education & Research, Ooty, India; ^3^Department of Pharmaceutical Biotechnology, JSS College of Pharmacy, JSS Academy of Higher Education & Research, Ooty, India

**Keywords:** benzohydrazide derivatives, phenylacetamide derivatives, ParE, antibacterial activity, antibiofilm activity

## Abstract

Antibacterial resistance (ABR) is a major life-threatening problem worldwide. Rampant dissemination of ABR always exemplified the need for the discovery of novel compounds. However, to circumvent the disease, a molecular target is required, which will lead to the death of the bacteria when acted upon by a compound. One group of enzymes that have proved to be an effective target for druggable candidates is bacterial DNA topoisomerases (DNA gyrase and ParE). In our present work, phenylacetamide and benzohydrazides derivatives were screened for their antibacterial activity against a selected panel of pathogens. The tested compounds displayed significant antibacterial activity with MIC values ranging from 0.64 to 5.65 μg/mL. Amongst 29 title compounds, compounds 5 and 21 exhibited more potent and selective inhibitory activity against *Escherichia coli* with MIC values at 0.64 and 0.67 μg/mL, respectively, and MBC at onefold MIC. Furthermore, compounds exhibited a post-antibiotic effect of 2 h at 1× MIC in comparison to ciprofloxacin and gentamicin. These compounds also demonstrated the concentration-dependent bactericidal activity against *E. coli* and synergized with FDA-approved drugs. The compounds are screened for their enzyme inhibitory activity against *E. coli* ParE, whose IC_50_ values range from 0.27 to 2.80 μg/mL. Gratifyingly, compounds, namely 8 and 25 belonging to the phenylacetamide series, were found to inhibit ParE enzyme with IC_50_ values of 0.27 and 0.28 μg/mL, respectively. In addition, compounds were benign to Vero cells and displayed a promising selectivity index (169.0629–951.7240). Moreover, compounds 1, 7, 8, 21, 24, and 25 (IC_50_: <1 and Selectivity index: >200) exhibited potent activity in reducing the *E. coli* biofilm in comparison with ciprofloxacin, erythromycin, and ampicillin. These astonishing results suggest the potential utilization of phenylacetamide and benzohydrazides derivatives as promising ParE inhibitors for treating bacterial infections.

## Introduction

Antibacterial resistance (ABR) is one of the significant public health concerns of the twenty-first century that threatens the prevention and treatment of an ever-increasing range of various pathogenic infections (Fischbach and Walsh, 2009; Silver, 2011; Michael et al., 2014). Though, WHO has taken many initiatives to combat ABR, lack of adequate surveillance in many parts leaves large gaps in existing knowledge for dissemination and extent of the ABR (Simpkin et al., 2017; Huttner et al., 2019; Piddock, 2019). Infections caused by resistant strains are speculated to cause millions of deaths in the near future unless suitable actions are taken to overwhelm the risk (Nathan, 2004; Davies and Davies, 2010; Roca et al., 2015). Thus, there is an urgent need to discover newer chemo-types with a novel mode of action to overcome the ABR (Miller and Waldrop, 2010). Among all the validated bacterial targets, DNA topoisomerases are considered efficient for the growth of bacterial cells and possess all the prerequisites of an “Ideal” target (Maxwell, 1997; Maxwell and Lawson, 2003). The ATP-dependent bacterial topoisomerases comprise DNA gyrase (Gyrase A and Gyrase B) and topoisomerase IV (ParC and ParE). These two enzymes play a vital role in DNA replication, leading to bacterial cell proliferation (Drlica and Zhao, 1997). DNA Gyrase belongs to the class II topoisomerase, a heterotetramer, involved in introducing negative supercoils into constrained DNA strands. Topoisomerase IV, primarily responsible for the decatenation of daughter chromatids at the end of replication (Heisig, 2001). Fluoroquinolone class of drugs is the first line of therapeutics to inhibit the catalytic subunits of DNA gyrase and topoisomerase IV. However, their use has been limited because of its side effects, toxicity, and emerging bacterial resistances (Jones, 1989; Booker et al., 2005).

GyrB and Topo IV (ParE), bacterial enzymes possess structural similarities in conserved regions, quinolones and coumarins sensitivity and, subunit organization. However, the most considerable difference between these enzyme structures is the long α-helices of each monomer’s C-terminus (Tourova et al., 2010). In *E. coli*, it is clear that fluoroquinolones preferentially bind to ParE enzyme than GyrB. Indeed, ParE is an unexplored target and could not establish any type of drug resistance. So, we concentrated on exploring the efficiency of small molecule inhibitors targeting ParE enzyme.

*E. coli* ParE ATPase catalytic 24-kDa domain (residues 1–217) consists of five α-helices and eight β-sheet strands along with a highly conserved amino acid sequence across the bacterial species. Novobiocin is an ATP-competitive inhibitor of the topoisomerases; however, activity is always greater for ParE than gyrase. This difference in the potency of novobiocin is due to a single residue (M74 in ParE) (Wigley et al., 1991). The ATP substrate binds to the catalytic pocket and is involved in hydrogen bond donating and accepting interactions between ligand and ParE crystal structure (Brino et al., 2000; Azam et al., 2015). These interactions are homologous to the donor/acceptor motif, explored in drug design and development of kinase inhibitors. Indeed, pyrrolopyrimidines, azaindoles, pyridylureas, quinazolidinediones series exploit these donor/acceptor interactions with the ParE protein.

In our earlier work, we reported the synthesis, minimum inhibitory concentration (MIC), minimum bactericidal concentration (MBC), time-kill kinetics of phenylacetamide and benzohydrazides derivatives (1–29) (Supplementary Table 1) (Yele et al., 2020, 2021). In continuation of our work, we report *in vitro* MIC and MBC against *Escherichia coli* (NCIM 2065) (*E. coli*), methicillin-resistant *Staphylococcus aureus* (MRSA) (NCIM 5021 and ATCC 43300). Whereas, time-kill kinetics, synergy testing, post-antibiotic effect (PAE) and anti-biofilm studies were performed against *E. coli*. Furthermore, we determined the IC_50_ values of title compounds (1–29) against *E. coli* ParE enzyme by malachite green assay.

## Materials and Methods

### *In vitro* Studies

#### Bacterial Strains and Media

The panel of bacteria selected for the current study are *Escherichia coli* (NCIM 2065) (*E. coli*), methicillin-resistant *Staphylococcus aureus* (MRSA) (NCIM 5021 and ATCC 43300). These strains were retrieved from the National Collection of Industrial Microorganisms (NCIM), Pune, and American Type Culture Collection repositories. All the strains were cultured using Mueller-Hinton broth before the study.

#### Antibacterial Susceptibility Test (AST) Against a Selected Panel of Organisms

AST was performed on the compounds (1–29) (Supplementary Table 1) (Yele et al., 2020, 2021) concerning the standard protocol (CLSI, 2017). The lowest concentration of a compound at which inhibition of bacterial growth was observed is termed MIC. The microbroth dilution method in Hi-media, Mueller Hinton Broth (MHB), was used to calculate the MIC. Bacterial cultures were diluted at a ratio of 1:1,000 MHB to achieve turbidity of 0.5 McFarland. DMSO and ciprofloxacin were used as a negative and positive control, respectively. The bacterial inoculum was added into a series of 96-well plates containing various test compounds ranging from 0.625 to 20 μg/mL. Plates were incubated for 20–24 h at 37°C. The absorbance was recorded at 600 nm of wavelength using Tecan-i-control, 1.7.1.12. MIC determinations for each compound were carried out in triplicate using a duplicate sample each time (Eloff, 1998; Agyare et al., 2012).

#### Minimum Bactericidal Concentration (MBC) Against a Selected Panel of Organisms

MBC can be defined as the minimum concentration of a compound required to kill the bacteria over a fixed period under precise conditions. MBC was determined for the synthesized compounds (1–29) (Supplementary Table 1; Yele et al., 2020, 2021) concerning the standard protocol (CLSI, 2017). Three successive concentrations above the MIC were employed, and 20 μL of the aliquot were sub-cultured onto the prepared nutrient agar plates, followed by incubation at 37°C for 24 h. Colonies were counted, and colony forming unit (CFU) per milliliter was calculated. MBC value is the concentration at which the ≥99% reduction (3 log 10) of bacterial growth is observed (Ahmad et al., 2015).

#### Time-Kill Kinetics

Bactericidal activity was evaluated for the compounds by the time-kill kinetics. The bacterial strain *E. coli* were diluted up to ∼10^6^ μg/mL and treated with a compound of concentration 1× and 0.5× MIC of compounds 5 and 21 and ciprofloxacin in MHB and incubated at 37°C. 50 μL aliquot of the culture was inoculated at regular time intervals of 0, 2, 4, 8, 16, and 24 h onto the solidified agar plates, followed by incubation at 37°C for 24 h. Ciprofloxacin is used as a reference throughout the study. CFU was calculated by the colonies formed on the agar plate at each time interval. Time-kill curves were plotted using the CFU/mL of surviving bacteria at each time point in the absence or presence of the compound (Tsuji et al., 2008).

#### Determination of Synergy With FDA Approved Drugs

The checkerboard method^1^ was used to determine the synergy between the test sample and the antibiotics (ciprofloxacin, gentamicin, rifampicin and doxycycline) against a panel of bacteria (Pandey et al., 2017). Serial twofold dilutions of each antibiotic to at least double the MIC were freshly prepared before testing. According to CLSI guidelines, the test compounds were serially diluted along the ordinate ranged from 0.625 to 20 μg/mL. In comparison, the antibiotics were serially diluted as shown along the abscissa ranged from 0.625 to 5 μg/mL/ml in 96 well microtiter plate. An inoculum ∼10^6^ CFU/mL of specific bacterial strain was prepared, and 100 μL was inoculated in each microtiter well, and the plates were incubated at 37°C for 24 h under aerobic conditions. The ΣFICs (fractional inhibitory concentrations) were calculated as follows:

$$\Sigma \,F\,I\,C=F\,I\,C\,A+F\,I\,C\,B=\frac{A}{M\,I\,C\,A}+\frac{B}{M\,I\,C\,B}$$

Where, A and B refers to the MIC of each antibiotic in combination (in a single well), and MICA and MICB are the MIC of each drug individually. FIC A is the MIC of drug A in the combination/MIC of drug A alone, and FIC B is the MIC of drug B in the combination/MIC of drug B alone. The combination is considered synergistic when the ΣFICs is ≤ 0.5, indifferent when the ΣFIC is > 0.5–4, and antagonistic when the ΣFICs is > 4 (Vogelman and Craig, 1985; Domínguez et al., 2001; Odds, 2003; Odenholt et al., 2003).

#### Determination of Post Antibiotic Effect (PAE)

To determine the PAE, young culture of *E. coli* was diluted in MHBII ∼10^6^ CFU/mL and exposed to 0.5× and 1× MIC of ciprofloxacin, gentamicin, and compounds 5 and 21 incubated at 37°C for 1 h (Suller and Lloyd, 2002). The culture was centrifuged and washed twice with MHBII to remove the traces of antibiotics used. The cells obtained were resuspended in antibiotic or sample free MHBII and incubated at 37°C. Samples collected for every 1 h were inoculated on the agar plate followed by the enumeration of CFU. PAE can be calculated using the following equation:

P⁢A⁢E=T-C

T refers to difference in time required for 1 Log 10 increase in CFU vs. CFU observed immediately after removing the test sample or antibiotic. C refers to similarly treated sample free control.

#### Enzyme Inhibitory Activity of *E. coli* ParE Enzyme

*In vitro* enzyme, the inhibitory assay was performed for the synthesized compounds 1–29 using a commercial ParE assay kit (ProFoldin, Hudson, United States). The assay was performed as per the standard protocol provided by the ProFoldin. The title compounds were screened at six concentrations ranging from 0.1 to 3.2 μg/mL. ParE hydrolyzes ATP as the source of molecular energy to perform decatenation reactions. Fluoroquinolones and aminocoumarins usually block ATPase activity to hinder the biological activities of topoisomerase II. Thus, the topoisomerase ATPase assay can be used for high-throughput screening of topoisomerase inhibitors. The ATPase activity of the *E. coli* ParE enzyme was based on the detection of inorganic phosphate produced by the hydrolysis of ATP in the presence of DNA. The phosphate is detected using a microplate reader at an absorbance wavelength of 650 nm. The selected compounds were tested for the inhibition of ATPase activity of ParE enzyme. The total volume of ATPase assay reaction mixture consists of 18 μL of H_2_O, 3 μL of 2 mM ATP, 3 μL of 10 × Buffer, 3 μL of 10 × DNA, 3 μL of 10 × *E. coli* ParE and different concentrations of the test sample were dissolved in DMSO. The reaction mixture was incubated for 1 h at 37°C and then quenched with 45 μL of Dye. Incubate for 5 min, and the absorbance was measured at 650 nm. The IC_50_ values were determined from the absorbance readings using no enzyme and no compound controls. They were calculated using the non-linear regression curve fit method.

#### *In vitro* Cytotoxicity Assay

Vero cells were centrifuged, and cell count was adjusted to ∼10^5^ cells/mL using Hi-media, Dulbecco’s Modified Eagle Medium (DMEM) containing 10% FBS. 100 μL (approximately 10,000 cells/well) of the diluted cell suspension was added to each well of a 96-well plate and incubated 24 h (Patel et al., 2009). Then the cells were centrifuged, obtained pellets were resuspended with 100 μL of different concentrations of test samples prepared in maintenance media and incubated at 37°C for 48 h in a 5% CO_2_ atmosphere. Microscopic examination of plates was carried out, and observations were recorded every 24 h. 20 μL of 3-(4,5 dimethyl thiazole-2-yl)- 2,5-diphenyl tetrazolium bromide (MTT, 2 mg/mL) was added, and plates were gently shaken and incubated for 2 h at 37°C in 5% CO_2_ atmosphere. To solubilize the formed formazan crystals, 100 μL of DMSO was added and gently shaken. The absorbance was measured using a microplate reader at a wavelength of 540 nm. The % of cell viability was calculated, and the concentration of drug or test samples required to inhibit the cell growth by 50% was generated from the dose-response curves.

#### Determination of *E. coli* Antibiofilm Activity of the Compound

*E. coli* (NCIM 2065) were grown overnight in 1% tryptic soy broth (TSB) medium at 37°C, followed by dilution (1:100) in fresh TSB medium. The freshly diluted culture (200 μL) was transferred into 96 well plates, covered with adhesive foil for maintaining low oxygen inside the plate and incubated for 48 h at 37°C in static condition. The media was decanted, and the plate was rinsed gently with phosphate buffer saline (PBS) (pH 7.4) to remove planktonic *E. coli*. Plates were refilled with different drug concentrations prepared in TSB and incubated at 37°C for 24 h. The media was decanted, washed thrice with PBS, and fixation of biofilm was achieved after incubating the plate at 60°C for 1 h. The formed biofilm is stained using 0.06% crystal violet for 10 min, washed with PBS, and dried at room temperature. Biofilm quantification was carried out by eluting the crystal violet using 30% acetic acid (200 μL) (Kwasny and Opperman, 2010). The absorbance was recorded at 600 nm of wavelength using Tecan-i-control, 1.7.1.12.

### Computational Studies

#### Molecular Docking

The 3D-structures of 29-compounds and X-ray crystallized protein structure were prepared using LigPrep available in Maestro (Schrödinger suite). Further, low energy conformers were selected for docking. The X-ray crystal structure of protein was prepared using Protein preparation wizard available in Maestro (Schrödinger suite) (Sastry et al., 2013). Hydrogens were added and water molecules were deleted in the protein crystal structures using Epik module. Addition of missing side chains and loops followed by generation of tautomeric states and protonation of amino acids using the module Prime (Jacobson et al., 2004). Protein minimization was carried out with a RMSD of 0.30 Å for crystallographic heavy atoms using OPLS3e forcefield (Roos et al., 2019). Furthermore, a grid box was generated at the centroid of active site (co-crystallized ligand) keeping the partial charge cutoff and van der Waals scaling at 0.25 and 1 Å, respectively. The obtained conformers from LigPrep were docked using Glide in extra-precision (XP) mode without smearing any constraints (Friesner et al., 2006). The best-docked poses of ligands were selected based on the Glide emodel, Glide Gscore, and Glide energy.

#### Binding Free Energy Calculations Using MMGBSA Approach

The BFG of synthesized ligands were computed using molecular mechanics-generalized born surface area (MM-GBSA)/Prime approach with OPLS3e forcefield (Roos et al., 2019). Energy minimization of protein-ligand complexes and simulation were carried out using Prime and VSGB 2.0 energy model (Li et al., 2011), respectively. Optimized implicit solvation model and physics-based corrections included in this energy model for all types of interactions (Hydrogen bonding, self-contact, hydrophobic, π-π stacking, π-cationic interactions).

### Statistical Analysis

Mean absorbances and their standard deviations (SDs) were computed for tested strains and controls, determined in triplicate and repeated three times. A two-way analysis of variance was used to determine the statistical significance. Statistical analysis was performed in GraphPad Prism v5.01 software (San Diego, CA, United States). A *p*-value ≤ 0.01 or lower was considered as statistically significant.

## Results

### *In vitro* Studies

#### Antibacterial Susceptibility Test (AST) Against a Selected Panel of Organisms

AST of selected compounds was determined using the micro-broth dilution method according to CLSI (2017). The MIC of the selected compounds was determined in the concentration range of 0.625–20 μg/mL against *E. coli* and MRSA strains. The MIC of compounds against other organisms was already reported (Yele et al., 2020, 2021). The obtained MIC values are compared with standard drug ciprofloxacin, and the results are given in Table 1.

**TABLE 1:** Minimum inhibitory concentration (MIC) of synthesized compounds (1–29) against the selected bacterial strains.

**Compound**	**Minimum inhibitory concentration (μg/mL)**^¶^		
	***E. Coli* (NCIM 2065)^*a*^**	**MRSA (NCIM 5021)^*b*^**	**MRSA (ATCC43300)^*c*^**
1	0.72 ± 0.07	0.84 ± 0.13	0.99 ± 0.24
2	1.01 ± 0.19	2.41 ± 0.65	1.34 ± 0.49
3	1.81 ± 0.15	1.84 ± 0.13	1.79 ± 0.21
4	2.49 ± 0.31	2.69 ± 0.49	2.52 ± 0.37
5	0.64 ± 0.03	0.72 ± 0.05	0.68 ± 0.08
6	1.56 ± 0.30	1.86 ± 0.10	1.71 ± 0.21
7	0.83 ± 0.05	0.95 ± 0.06	0.85 ± 0.09
8	0.93 ± 0.03	0.66 ± 0.13	1.06 ± 0.12
9	1.64 ± 0.10	1.71 ± 0.16	1.75 ± 0.21
10	0.83 ± 0.05	0.82 ± 0.03	0.88 ± 0.10
11	0.97 ± 0.01	1.79 ± 0.16	0.84 ± 0.05
12	0.86 ± 0.03	2.65 ± 0.46	0.96 ± 0.14
13	2.60 ± 0.26	8.36 ± 0.30	2.47 ± 0.46
14	4.14 ± 0.07	4.35 ± 0.09	0.94 ± 0.14
15	0.98 ± 0.07	5.60 ± 0.36	1.51 ± 0.49
16	2.60 ± 0.28	5.65 ± 0.27	2.74 ± 0.19
17	3.41 ± 0.25	3.71 ± 0.15	3.23 ± 0.21
18	1.42 ± 0.23	1.81 ± 0.40	1.74 ± 0.21
19	2.06 ± 0.14	2.67 ± 0.11	2.38 ± 0.16
20	4.70 ± 0.41	4.94 ± 0.05	4.92 ± 0.05
21	0.67 ± 0.02	0.68 ± 0.005	0.70 ± 0.06
22	0.89 ± 0.01	0.90 ± 0.04	0.76 ± 0.06
23	0.95 ± 0.05	0.94 ± 0.04	0.75 ± 0.11
24	0.78 ± 0.08	0.81 ± 0.10	0.73 ± 0.12
25	0.72 ± 0.05	0.86 ± 0.13	1.11 ± 0.12
26	2.64 ± 0.31	2.77 ± 0.21	2.78 ± 0.20
27	1.59 ± 0.38	1.71 ± 0.24	1.06 ± 0.14
28	1.88 ± 0.11	2.70 ± 0.22	2.04 ± 0.16
29	0.81 ± 0.14	0.75 ± 0.19	0.91 ± 0.11
Ciprofloxacin	0.62 ± 0.015	0.67 ± 0.02	0.70 ± 0.03

*^¶^ Values are mean ± SEM (*n* = 3); Positive control = Ciprofloxacin.*

*^*a*^*E. coli*, *Escherichia Coli* (NCIM 2065).*

*^*b*^MRSA, Methicillin resistant *Staphylococcus aureus* (NCIM 5021).*

*^*c*^MRSA, Methicillin resistant *Staphylococcus aureus* (ATCC 43300).*

All compounds exhibited good antibacterial activity with MIC values ranging from 0.64 to 5.65 μg/mL. However, it should be noticed that the sensitivity of bacterial strains toward title compounds was in general different. The compounds 5 and 21 appeared to be most active against *E. coli* with an MIC of 0.64 and 0.67 μg/mL followed by compound 1 (MIC, 0.72 μg/mL), 24 (0.78 μg/mL) and 25 (0.72 μg/mL).

In addition, compounds 8 (0.66 μg/mL) and 21 (0.68 μg/mL) showed significant antibacterial activity against MRSA (NCIM 5021). While MRSA (ATCC 43300) appeared to be sensitive against compound 5 with MIC at 0.68 μg/mL.

#### Minimum Bactericidal Concentration (MBC) Against a Selected Panel of Organisms

MBC is the minimum concentration of a compound required to kill 99.9% of bacteria. The MBC/MIC ratio of ≤2 demonstrates that the compound has bactericidal and tolerance development if the ratio is ≥4. The MBC of the selected compounds was tested against *E. coli* and MRSA strains. The MBC of compounds against other organisms was already reported (Yele et al., 2020, 2021). All the compounds exhibited significant bactericidal activity against *E. coli* and MRSA strains (Table 2).

**TABLE 2:** Minimum bactericidal concentration (MBC) (μg/mL)^¶^ and MBC/MIC ratio of benzohydrazides and phenylacetamide derivatives (1–29) against a panel of organisms.

**Compound**	***E. Coli***		**MRSA**		**MRSA**	
	**(NCIM 2065)^*a*^**		**(NCIM 5021)^*b*^**		**(ATCC43300)^*c*^**	
	**MBC**	**MBC/MIC**	**MBC**	**MBC/MIC**	**MBC**	**MBC/MIC**
1	1.42 ± 0.02	2	0.84 ± 0.13	1	0.99 ± 0.24	1
2	2.00 ± 0.01	2	4.8 ± 0.21	2	2.71 ± 0.21	2
3	1.75 ± 0.09	1	1.84 ± 0.13	1	1.79 ± 0.21	1
4	4.71 ± 0.03	2	8.00 ± 0.04	3	8.76 ± 0.21	3
5	0.64 ± 0.42	1	1.54 ± 0.11	2	0.68 ± 0.08	1
6	3.7 ± 0.12	3	7.39 ± 0.18	4	4.56 ± 0.08	3
7	0.83 ± 0.1	1	0.95 ± 0.06	1	0.85 ± 0.09	1
8	0.93 ± 0.03	1	0.63 ± 0.13	1	2.46 ± 0.12	2
9	3.68 ± 0.29	2	4.70 ± 0.41	3	6.29 ± 0.08	4
10	0.83 ± 0.05	1	0.82 ± 0.03	1	0.88 ± 0.10	1
11	0.97 ± 0.01	1	7.18 ± 0.15	4	0.84 ± 0.05	1
12	0.86 ± 0.03	1	4.4 ± 0.15	2	0.96 ± 0.14	1
13	9.43 ± 0.11	4	–	–	–	–
14	0.98 ± 0.07	1	–	–	0.94 ± 0.14	1
15	–	–	–	–	3.18 ± 0.32	2
16	9.12 ± 0.11	4	–	–	–	–
17	7.2 ± 0.7	2	7.46 ± 0.05	2	–	–
18	1.42 ± 0.23	1	1.81 ± 0.40	1	3.74 ± 0.16	2
19	2.06 ± 0.14	1	5.37 ± 0.11	2	4.74 ± 0.16	2
20	4.59 ± 0.26	1	–	–	–	–
21	0.67 ± 0.02	1	0.68 ± 0.005	1	2.16 ± 0.30	3
22	0.89 ± 0.01	1	0.90 ± 0.04	1	0.76 ± 0.06	1
23	0.95 ± 0.05	1	0.94 ± 0.04	1	0.75 ± 0.11	1
24	0.78 ± 0.08	1	0.81 ± 0.10	1	0.73 ± 0.12	1
25	7.09 ± 0.02	3	8.37 ± 0.17	3	–	–
26	0.72 ± 0.05	1	0.86 ± 0.13	1	4.32 ± 0.10	4
27	1.59 ± 0.38	1	3.55 ± 0.07	2	1.06 ± 0.14	1
28	3.85 ± 0.25	2	10.62 ± 0.44	4	–	–
29	0.81 ± 0.14	1	0.75 ± 0.19	1	0.91 ± 0.11	1
Cipro	1.42 ± 0.02	2	0.84 ± 0.13	1	0.99 ± 0.24	1

*^¶^ Values are mean ± SEM (*n* = 3).*

*“–”: not determined; Positive control = Ciprofloxacin.*

*^*a*^*E. coli*, *Escherichia Coli* (NCIM 2065).*

*^*b*^MRSA, Methicillin resistant *Staphylococcus aureus* (NCIM 5021).*

*^*c*^MRSA, Methicillin resistant *Staphylococcus aureus* (ATCC 43300).*

#### Time-Kill Kinetics

**FIGURE 1:**
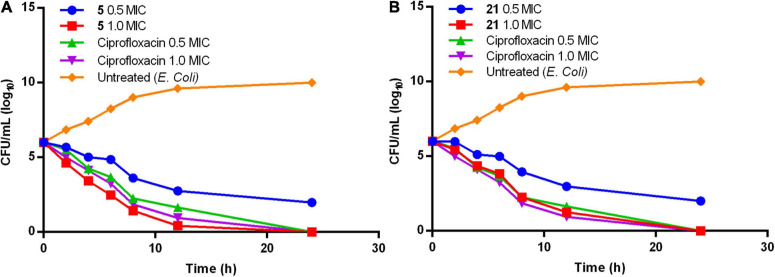
Time-kill kinetics of compound **(A)** **5** and **(B)** **21**.

Compounds 5 and 21 exhibited significant MIC (0.64 and 0.67 μg/mL), and MBC values at onefold MIC against *E. coli* are considered for further evaluation. Time-kill kinetics demonstrated the bactericidal property of compounds 5 (Figure 1A) and 21 (Figure 1B) against *E. coli*. The *E. coli* bacterial culture was serially diluted to attain ∼10^5^ CFU/mL concentration and treated with test samples and ciprofloxacin at concentrations of 0.5× and 1.0× MIC. The 50 μL was collected and sub-cultured on an agar plate at regular time intervals of 0, 2, 4, 8, 16, and 24 h, respectively. The cultured agar plates were incubated at 37°C. CFU was calculated from the colonies formed on the agar plate at each time interval. Time-kill curves were plotted using the CFU/mL of surviving bacteria at each time point in the compound’s absence or presence. Tested samples exhibited significant bactericidal property against *E. coli* compared to the untreated *E. coli* and culture with ciprofloxacin.

#### Determination of Synergy With FDA Approved Antibiotics

The checkerboard method was employed to determine the synergy between compounds 5, 21, and antibiotics ciprofloxacin, gentamicin, rifampicin and doxycycline. The results have shown that compounds 5 (Table 3) and 21 (Table 4) synergized with the FDA-approved antibiotics, demonstrating significant potential to be a part of a multi-drug regimen.

**TABLE 3:** Determination of synergy of compound 5 with approved antibiotics.

**Drug**	***E. coli***	**MIC of compound 5 in the presence of the drug (A)**	**MIC of the drug in the presence of compound 5 (B)**	**FIC A**	**FIC B**	**ΣFIC A + FIC B**	**Inference**
Compound 5	0.64						
Ciprofloxacin	0.62	0.0996	0.09652	0.1606	0.1556	0.3162	Synergistic
Gentamicin	0.52	0.0756	0.06523	0.1453	0.1254	0.2693	Synergistic
Rifampicin	0.49	0.0589	0.02569	0.1202	0.0524	0.1726	Synergistic
Doxycycline	0.36	0.0253	0.00986	0.0702	0.0273	0.0975	Synergistic

**TABLE 4:** Determination of synergy of compound 21 with approved antibiotics.

**Drug**	***E. coli***	**MIC of compound 21 in the presence of the drug (A)**	**MIC of the drug in the presence of compound 21 (B)**	**FIC A**	**FIC B**	**ΣFIC A + FIC B**	**Inference**
Compound 21	0.67						
Ciprofloxacin	0.62	0.1256	0.09852	0.2025	0.1589	0.3614	Synergistic
Gentamicin	0.52	0.0992	0.07856	0.1907	0.1510	0.3417	Synergistic
Rifampicin	0.49	0.0952	0.06278	0.1942	0.1281	0.3223	Synergistic
Doxycycline	0.36	0.0653	0.00945	0.1813	0.0262	0.2075	Synergistic

#### Determination of Post-antibiotic Effect (PAE) of Compounds 5 and 21

PAE of compounds 5 and 21 were evaluated with ciprofloxacin and gentamicin as controls. The results demonstrated that compounds 5 and 21 exhibited a PAE of ∼2 h at 10× MIC compared to ciprofloxacin and gentamicin (Table 5).

**TABLE 5:** Post-antibiotic effect of compound 5 and 21.

**Treatments**	**Time for 1 log10 (h)**	**PAE = T - C**
*E. coli*	2	0
Compound **5** 1 × mic	3	1
Compound **5** 10 × mic	4	2
Compound **21** 1 × mic	4	2
Compound **21** 10 × mic	4	2
Ciprofloxacin 1 × mic	3	1
Ciprofloxacin 10 × mic	4	2
Gentamicin 1 × mic	2	0
Gentamicin 10 × mic	3	1

#### Enzyme Inhibition Assay of *E. coli* ParE Enzyme

**FIGURE 2:**
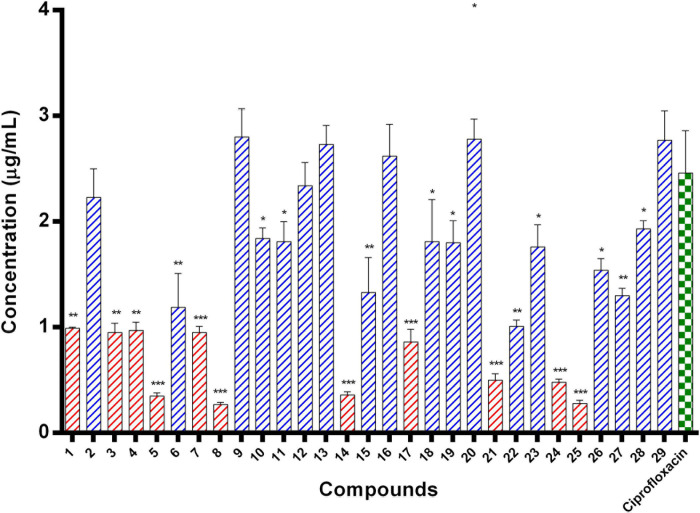
IC_50_ values of 29 compounds against *E. coli* ParE enzyme. Data are expressed as mean ± SD (*n* = 3). *p ≤ 0.05, **p ≤ 0.005, ***p ≤ 0.001.

**FIGURE 3:**
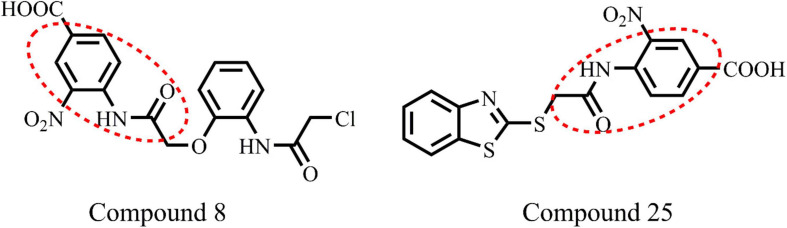
Chemical structure of highly active compounds 8 and 25 in phenylacetamide series.

*E. coli* ParE enzyme inhibition assay was performed for the synthesized compounds 1–29 using commercial ParE assay kit (ProFoldin, Hudson, United States). The compounds displayed IC_50_ values (Figure 2) in the range of 0.27–2.80 μg/mL. Among, the assayed molecules, the two compounds namely 8 and 25 belonging to the phenylacetamide series were found to inhibit ParE enzyme with IC_50_ values of 0.27 and 0.28 μg/mL, respectively (Figure 3). The other compounds 1 (0.99 μg/mL), 3 (0.95 μg/mL), 4 (0.95 μg/mL), 5 (0.35 μg/mL), 7 (0.95 μg/mL), 14 (0.36 μg/mL) 17 (0.86 μg/mL), 21 (0.50 μg/mL), and 24 (0.48 μg/mL) belonging to both phenylacetamide and benzohydrazides series displayed significant inhibitory activity against ParE enzyme with IC_50_ values less than <1 μg/mL. The compounds 6 (1.19 μg/mL), 10 (1.84 μg/mL) 11 (1.81 μg/mL), 15 (1.33 μg/mL), 18 (1.81 μg/mL), 19 (1.80 μg/mL), 22 (1.01 μg/mL), 23 (1.76 μg/mL), 26 (1.54 μg/mL), 27 (1.30 μg/mL), and 28 (1.93 μg/mL) exhibited variable activity against *E. coli*ParE enzyme with IC_50_ values ranging from 1 to 2 μg/mL. Only few compounds 2 (2.33 μg/mL), 9 (2.80 μg/mL), 12 (2.34 μg/mL), 13 (2.73 μg/mL), 16 (2.62 μg/mL), 20 (2.78 μg/mL), and 29 (2.77 μg/mL) displayed partial significant inhibition against ParE enzyme with IC_50_ values in the range of 2–2.80 μg/mL.

#### *In vitro* Cytotoxicity Assay

The compounds whose IC_50_ is <1 μg/mL were tested for cytotoxicity profile against Vero cells using MTT assay. CC_50_ is referred to as the lowest concentration of the compound, leads to a 50% cell viability reduction with doxorubicin as a positive control. In addition, the selectivity index (SI) was also calculated by dividing CC_50_ with antibacterial activity. Table 6 explains that compounds are non-toxic to Vero cells (61.7583–256.9655) and exhibited a selectivity index (169.0629–951.7240).

**TABLE 6:** Cytotoxicity profile against Vero cells and Selectivity index (SI) of selected compounds against *E. coli*.

**Compounds**	**IC_50_ (μg/mL)^*a*^**,**^¶^**	**CC_50_ (μg/mL)^*b*^**,**^¶^**	**SI (CC_50_/IC_50_)^*c*^**
1	0.99 ± 0.01	213.9002	216.0608
3	0.95 ± 0.09	178.8742	188.2886
4	0.97 ± 0.08	163.9911	169.0629
5	0.35 ± 0.03	61.7583	176.4531
7	0.95 ± 0.06	224.7306	236.5585
8	0.27 ± 0.02	256.9655	951.7240
14	0.36 ± 0.03	64.7605	179.8902
17	0.86 ± 0.12	150.8056	175.3553
21	0.50 ± 0.06	170.9509	341.9018
24	0.48 ± 0.03	168.8089	351.6852
25	0.28 ± 0.03	193.9775	692.7767

*^¶^ Values are mean ± SEM (*n* = 3).*

*^*a*^IC_50_, Inhibitory concentration.*

*^*b*^CC_50_, Cell cytotoxicity.*

*^*c*^SI, Selectivity index.*

#### Determination of Antibiofilm Activity of Compounds

**FIGURE 4:**
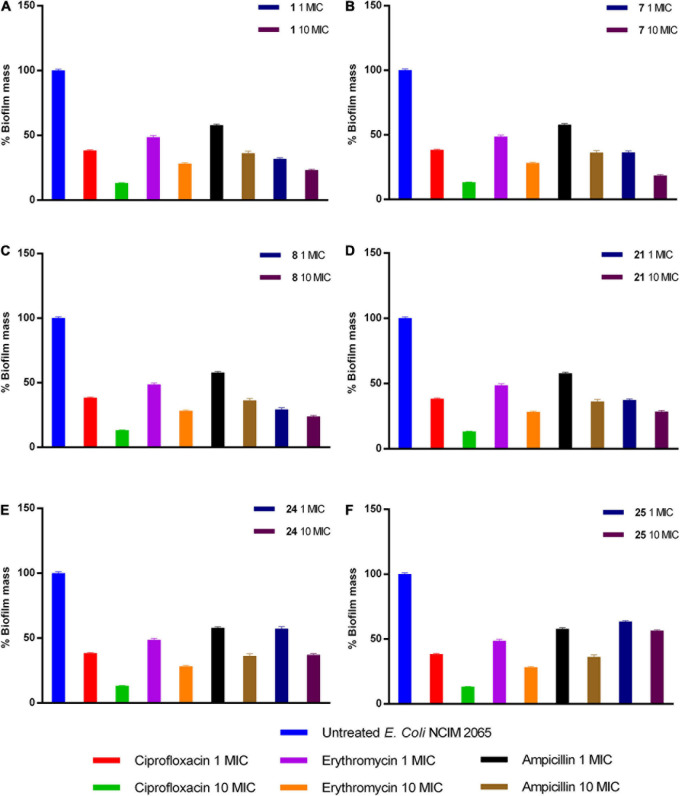
Activity of **(A)** 1, **(B)** 7, **(C)** 8, **(D)** 21, **(E)** 24, and **(F)** 25 compounds against preformed *E. coli* biofilm.

Upon analysis of results obtained through enzyme inhibition assay and selectivity index, we selected compounds 1, 7, 8, 21, 24, and 25 (IC_50_: <1 and Selectivity index: >200) for *E. coli* antibiofilm activity. The compound 1, 7, 8, 24, and 25 at 10× MIC displayed potent anti-biofilm activity in reducing biofilm by 76, 81, 76, 71, 62, and 43%, respectively. On the other hand, a similar reduction in biofilm was achieved at 10 × MIC of ciprofloxacin (86% reduction) and erythromycin (71% reduction), while ampicillin reduced only 63%. Thus, compounds 1, 7, 8, and 21 are more potent in reducing biofilm than ampicillin (Figure 4).

### Computational Studies

#### Molecular Docking Analysis

The ligand binds to the protein crystal structure with high binding affinity (high negative glide Gscore). The results of XP-molecular docking were provided in Table 7. The Gscore of all the compounds were found in the range of –2.822 to –5.375 kcal/mol. XP-docked poses of the compounds exhibited significant interactions like hydrogen bonding, self-contact, hydrophobic, π-π stacking, π-cationic interactions with conserved N-terminal domain residues Asp45, Asp69, Arg72, Gly73, His79, Ala86, Ile90, Ile116, and Ser117 (Supplementary Figures 1–29). The results were compared by redocking of the co-crystal into the binding pocket of *E. coli* ParE. The Gscore of co-crystal was found to be –7.603 kcal/mol. Further, the high active compounds 8 (Figure 4A) and 25 (Figure 4B) exhibited significant inhibitory activity as evident by their Gscore –5.412 and –5.375 kcal/mol.

**TABLE 7:** XP-docking and binding free energy (kcal/moL) results of synthesized compounds (1–29) against *E. coli* ParE enzyme (*Pdb.*3FV5).

**Compound**	**G_*score*_^a^**	**G_*evdw*_^b^**	**G_*ecoul*_^c^**	**G_*energy*_^d^**	**G_*emodel*_^e^**	**ΔG_*bind*_^f^**	**ΔG_*cou*_^g^**	**ΔG_*cov*_^h^**	**ΔG_*lipo*_^i^**	**ΔG_*vdW*_^j^**
1	–4.308	–36.835	–5.213	–42.049	–55.788	–52.95	–4.58	3.21	–14.2	–44.96
2	–3.807	–38.227	–0.989	–39.215	–51.384	–49.77	–3.35	2.01	–16.32	–40.56
3	–4.3	–29.727	–2.501	–32.228	–42.538	–54.77	–23.39	11.12	–17.86	–48.42
4	–4.06	–32.934	–8.172	–41.106	–53.746	–51.25	8.25	–0.74	–12.79	–41.63
5	–5.102	–41.29	–5.024	–46.314	–63.354	–59.01	–21.13	7.48	–14.06	–46.55
6	–3.82	–38.928	–0.114	–39.042	–50.53	–36.23	57.42	–3.18	–13.71	–46.88
7	–4.174	–36.44	–5.049	–41.489	–54.176	–56.03	–12.08	5.53	–14.81	–46.81
8	–5.412	–34.045	–9.903	–43.948	–57.427	–55.46	–2.29	–3.72	–11.76	–41.77
9	–3.974	–30.284	–4.935	–35.219	–40.157	–42.02	6.3	–1.01	–10.69	–31.31
10	–3.80	–29.717	–7.723	–37.441	–49.119	–49.47	–23.63	1.75	–12.49	–37.12
11	–4.00	–34.282	–6.275	–40.556	–54.34	–34.26	20.32	0.21	–11.43	–39.54
12	–3.90	–32.275	–3.585	–35.86	–44.883	–36.04	21.5	–7.63	–14.81	–37.93
13	–4.96	–38.729	–6.116	–44.846	–53.356	–40.83	14.5	–3.05	–15.71	–48.78
14	–5.23	–40.294	–6.191	–46.484	–64.091	–58.68	–18.03	6.28	–14.06	–39.52
15	–3.753	–31.978	–6.313	–38.291	–49.145	–46.92	0.76	2.47	–13.16	–40.34
16	–3.197	–27.948	–6.804	–34.752	–45.024	–49.76	6.06	–3.71	–16.41	–52.7
17	–5.088	–31.422	–11.556	–42.978	–51.123	–53.98	16.8	4	–12.26	–46.1
18	–3.465	–33.163	–3.935	–37.098	–49.193	–36.06	11.18	2.2	–11.73	–45.02
19	–3.7	–34.667	–3.595	–38.263	–47.487	–45.46	16.13	–10.32	–9.66	–39.12
20	–2.882	–41.022	–1.271	–42.294	–57.745	–46.21	–11.97	5.69	–13.36	–55.28
21	–5.047	–36.465	–7.82	–44.285	–61.693	–52.21	18.49	–5.01	–13.61	–43.41
22	–4.321	–27.6	–2.337	–29.937	–35.212	–48.07	12.82	–1.37	–15.59	–49.89
23	–3.08	–32.608	–1.623	–34.231	–42.826	–47.14	18.75	–0.36	–13.88	–48.72
24	–4.98	–40.03	–3.82	–43.85	–57.457	–50.62	43.39	–5.7	–9	–46.5
25	–5.375	–32.261	–4.108	–36.368	–51.822	–55.2	–35.02	14.15	–19.3	–55.34
26	–3.921	–33.208	–6.346	–39.553	–51.236	–37.42	7.02	–2.24	–10.17	–40.33
27	–3.89	–27	–2.385	–29.385	–35.68	–49.86	39.9	–9.17	–16.02	–42.19
28	–3.233	–27.633	–3.095	–30.727	–39.853	–37.41	24.34	–10.06	–13.19	–34.82
29	–4.018	–36.835	–5.213	–42.049	–55.788	–49.95	–4.58	3.21	–14.2	–44.96
Co-crystal	–7.603	–45.989	–10.429	–56.417	–93.24	–54.4	28.04	–11.91	–10.88	–52.99

*^*a*^Glide score (kcal/mol).*

*^*b*^Glide van der Waals energy (kcal/mol).*

*^*c*^Glide ecoul (kcal/mol).*

*^*d*^Glide energy (kcal/mol).*

*^*e*^Glide emodel (kcal/mol).*

*^*f*^Free energy of binding (kcal/mol).*

*^*g*^Coulomb energy (kcal/mol).*

*^*h*^Covalent energy (kcal/mol).*

*^*i*^Hydrophobic energy (kcal/mol).*

*^*j*^van der Waals energy (kcal/mol).*

#### Binding Free Energy (BFG) Calculations

The BFG of compounds with *E. coli* ParE was computed using MM-GBSA/Prime approach. The ΔGbind values were observed in the range of –34.26 to –55.20 kcal/mol. All the energy terms of Prime calculations were represented in the Table 7. Amongst, all the prime energy terms, van der Waals energy was found to be in the range of –31.31 to –55.28 kcal/mol.

## Discussion

In this article, the synthesized phenylacetamide and benzohydrazides derivatives were screened for their ParE inhibitory activity. Further, the compounds displayed potent antibacterial activity against *E. coli* and MRSA strains.

Both phenylacetamide and benzohydrazides series exhibited optimal activity against selected strains (Table 1). Gratifyingly, most of the compounds shown potential inhibition of *E. coli* than MRSA strains. The compounds which exhibited potency against *E. coli* were observed to inhibit MRSA (ATCC43300) at MIC <1 μg/mL. The activity in the phenylacetamide series may be attributed to amide linkage (29) or the presence of nitro (1, 7, 8) and carboxyl (5, 8) groups on the phenyl ring. In addition, compounds (23, 24, 25) have shown promising inhibition of bacteria because of benzothiazole ring flanked by substituted phenyl ring via an amide linkage. Moreover, the compounds in the benzohydrazides series also shown prominent inhibitory activity that may be ascribed to the presence of formylformohydrazide (O = C-NH-NH-C = O) (12, 15) or benzothiazole ring, which is linked to the substituted phenyl rings through the formylformohydrazide (21, 22) group.

Both phenylacetamide and benzohydrazides series displayed potent bactericidal activity against selected pathogens. Besides, compounds 5 (0.64 μg/mL) and 21 (0.64 μg/mL) exhibited significant bactericidal property against *E. coli* at onefold MIC. However, these two compounds also exhibited significant bactericidal properties against MRSA strains. The activity of compound 5 may be attributed to substitutions, such as carboxyl and bromine groups on the phenyl ring and amide moiety flanked between the two aromatic rings. On the other hand, compound 21 belonging to the benzohydrazides series also exhibited potential bactericidal activity may be due to the presence of formylformohydrazide connecting benzothiazole ring and nitro substituted phenyl ring. Furthermore, time-kill kinetics of compounds 5 and 21 (Figure 1) revealed the potential bactericidal property against *E. coli* compared to the ciprofloxacin and untreated *E. coli*.

After analyzing the activity of these compounds, we performed synergy testing using a checkerboard assay with different approved drugs like ciprofloxacin, gentamicin, rifampicin, and doxycycline. The results obtained were astounding, exemplifying that the selected compounds 5 and 21 were considered the best candidates in multi-drug regimens (Tables 3, 4).

In addition, compounds exhibited significant PAE at 10× MIC in 2 h compared to ciprofloxacin. In contrast, both compounds exhibited more activity at 10× MIC in comparison with gentamicin (Table 5).

All the compounds were screened for *E. coli* ParE inhibitory activity using an assay kit. The results revealed that all the tested compounds displayed significant inhibitory activity against ParE with the IC_50_ values in the range of 0.27 and 0.28 μg/mL (Figure 2). Almost 50% of the compounds exhibited significant activity against *E. coli* ParE with IC_50_ <1 μg/mL. While 20% exhibited very potent activity with IC_50_ values <0.5 μg/mL. The promising inhibition of these compounds may be attributed to the electron-withdrawing groups on the phenyl ring at positions two and four. In the phenylacetamide series, the high active compounds, namely 8 and 25, exhibited potential inhibition of *E. coli* ParE at IC_50_ of 0.27 ± 0.02 μg/mL and 0.28 ± 0.03 μg/mL, respectively. Fortunately, both compounds share common functional groups, such as carboxyl at position four and nitro at position two on the phenyl ring. However, the two compounds are dissimilar at the other side of molecular structure, substituted phenoxy moiety (8) and 2-mercaptobenzothiazole group (25) (Figure 3). Further, compounds 5 and 24 displayed promising activity with IC_50_ values of 0.35 ± 0.03 and 0.48 ± 0.06 μg/mL, which may be attributed to the presence of electron-withdrawing groups, such as carboxyl and nitro at position four of the phenyl ring. The variation among the activities of both the compounds maybe because of the substituted phenoxy moiety present in compound 5. However, the replacement of electron-withdrawing groups with chlorine atoms shown detrimental to the inhibitory activity (10, 11).

In the benzohydrazides series, the compounds 14 and 21 shown significant inhibitory activity against *E. coli* ParE at IC_50_ of 0.36 ± 0.02 and 0.50 ± 0.06 μg/mL, respectively. The potent activity of these compounds may be because of the presence of electron-withdrawing group NO_2_ at position four of the phenyl ring. However, on the other side, the molecules possess different chemical moieties (substituted phenoxy moiety; 14 and 2-mercaptobenzothiazole group; 21) responsible for the variation in the activity. The compounds possessing electron-releasing groups (12, 13, 19, 20) have exhibited partial inhibitory activity against *E. coli* ParE.

Furthermore, the compounds whose IC_50_ is <1 μg/mL were tested for cytotoxicity profile against Vero cells using MTT assay using doxorubicin as the reference standard. A perusal of cytotoxicity data reveals that all compounds are non-toxic to Vero cells in the range of 61.7583–256.9655 μg/mL and displayed potential SI [>20; (169.0629 to 951.7240)]. The higher SI value indicates that the drug was more effective and safer during *in vivo* treatment (Table 6).

Besides, compounds, which displayed promising SI (>200), were selected for antibiofilm activity. Bacteria under different stresses make biofilm protect themselves, leading to prolonged therapeutic intervention and increased drug-resistance. All the approved antibiotics possess minimal activity against biofilm because of the altered physiological state; thus, it is essential to determine the antibiofilm activity of compounds under development. Results explained that compounds 1, 7, 8, 21, 24, and 25 exhibited potent activity in biofilm reduction of *E. coli* at 10× MIC. Except compounds 24 and 25, the remaining compounds exhibited greater potency in antibiofilm activity than ampicillin (Figure 4).

**FIGURE 5:**
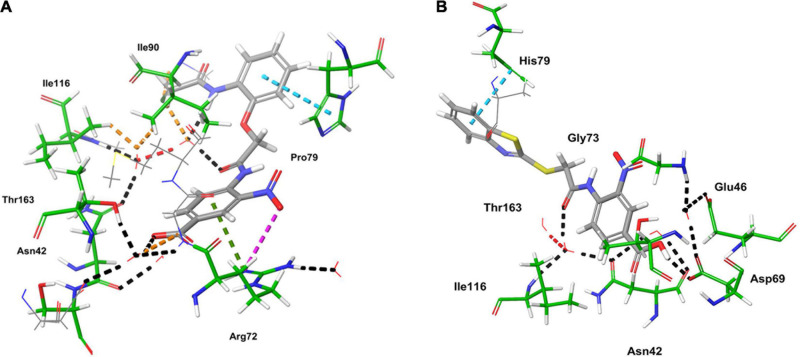
3D-interaction diagrams of **(A)** compound 8 and **(B)** compound 25 exposing key interactions with the catalytic residues of *E. coli* ParE enzyme.

Computational studies, such as molecular docking and binding free energy revealed better binding affinity of the compounds within the catalytic pocket of *E. coli* ParE (Table 7). From the Figure 5A that the active 8 (IC_50_ of 0.27 μg/mL) is placed well within the active site and occupied N-terminal domain of the pocket. It exhibited five hydrogen bonding interactions with Asn42, Gly73, Ile90, Ile116, Thr163 with carbonyl groups of the amide linker and carboxyl group. Further, benzene ring of compound formed a salt-bridge and π-cationic interactions with the protonated Arg72. Besides compound was stabilized by forming a π-π stacking interaction between the electron clouds of aromatic ring (compound 8) and imidazoline ring (His79). Like compound 8, another high active inhibitor 25 (0.28 μg/mL) (Figure 5B) also occupied N-terminal domain of the receptor and showed three hydrogen bonding interactions between the carbonyl groups of amide fragment and carboxylic acid with Asn42, Asp69, and Ile116. A salt bridge was also observed between the nitro group and protonated Arg72. Moreover, the compound stabilized by the π-π stacking interaction with His79. Similar interactions were observed from the docking pose of co-crystallized ligand (Supplementary Figure 30). The binding strength of the compounds 8 (57.427 kcal/mol) and 25 (57.822) indicated higher binding of the ligands in the catalytic pocket of enzyme. The non-polar solvation energy (ΔGsolv: –9.00 to 16.41 kcal/mol) term was moderately favorable while coulombs (ΔGcou: 43.39 to –23.63 kcal/mol) and covalent (ΔGcov: 11.12 to –17.86 kcal/mol) energy terms were unfavorable for the inhibitory activity. Further, it should be noted that van der Waals energy (ΔGvdW: –31.31 to –55.28 kcal/mol) term was found to be favorable and is the key driving forced for the ligand binding. This is in agreement with the Glide Emodel (–29.937 to –46.484 kcal/mol) having significant weighting of the force field.

## Structure Activity Relationship

**FIGURE 6:**
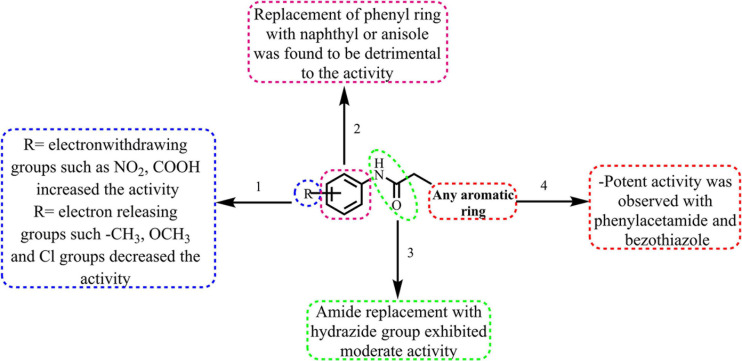
Broad structure-activity relationship of the phenylacetamide and benzohydrazides.

The broad structural activity relationship was summarized in Figure 6. Although, compounds have shown significant potential inhibition of *E. coli* ParE, the variation in their inhibitory concentrations might be because of the chemical substitutions at different positions on the aromatic rings.

(1) From the results, it is clear that compounds possessing electron-withdrawing groups, such as nitro and carboxyl moieties (8 and 25) increased the activity toward the *E. coli* ParE. However, electron releasing groups like methyl, methoxy groups decreased the inhibitory activity (12, 13, and 20). The halogen substitution on the aromatic rings possesses substantial activity of these compounds against *E. coli* ParE (11, 15, 28, and 29).
(2) Further, the replacement of the phenyl ring with fused aromatic system or with anisole markedly decrease the inhibitory activity (28 and 29).
(3) Both amide and hydrazide linker were found to be necessary for the activity as evident by their IC_5__0_s and docking results.
(4) Potent activity was observed with both the phenylacetamide and benzothiazole ring systems (8 and 25).

## Conclusion

We have previously synthesized and tested a series of phenylacetamide and benzohydrazides, which displayed interesting antibacterial activity against selected bacteria strains. In the current paper, we have successfully performed antibacterial screening for *E. coli* and MRSA strains. Amongst the title compounds, compounds 5 and 21 exhibited significant MIC and MBC. Further, these compounds showed PAE at 2 h compared to ciprofloxacin and gentamicin and established concentration-dependent bactericidal property. The compounds exhibited synergistic activity with FDA-approved drugs indicating the better potential for the multi-drug regimen. Besides, the molecular structures of the compounds 8 and 25 were found to be favorable for *E. coli* ParE inhibition as evident by their IC_50_ values IC_50_ of 0.27 ± 0.02 μg/mL and 0.28 ± 0.03 μg/mL, respectively. The significant values of CC_50_ and selectivity index indicated that drugs were more effective and safer during *in vivo* treatment.

Furthermore, compounds 8 and 25 also exhibited significant antibiofilm activity, as evidenced by their biofilm mass reduction. In summary, the phenylacetamide series (compounds 8 and 25) represent one of the most exciting chemical scaffolds in the bacterial armamentarium. As a novel chemical that addressing ABR by targeting unexplored targets like ParE, the representative compounds 8 and 25 can further be investigated to develop potent antibacterial agents for use against nosocomial pathogens.

## Data Availability Statement

The original contributions presented in the study are included in the article/Supplementary Material, further inquiries can be directed to the corresponding author/s.

## Author Contributions

VY synthesized the literature, drafted the manuscript, and revised the manuscript. BS and AW were involved in the *in vitro* studies. AM provided the conceptual inputs. All authors read and approved the final manuscript.

## Conflict of Interest

The authors declare that the research was conducted in the absence of any commercial or financial relationships that could be construed as a potential conflict of interest.

## Footnotes

1 https://emerypharma.com/biology/antimicrobial-synergy-study-checkerboard-assay/
