# Supplementary material for: Benzohydrazide and Phenylacetamide Scaffolds: New Putative ParE Inhibitors
Source: Front Bioeng Biotechnol. 2021 Jun 17;9:669728. doi: 10.3389/fbioe.2021.669728 (PMC8247773; doi:10.3389/fbioe.2021.669728)
Supplement: Supplementary file 1 [file Data_Sheet_1.PDF]

Supplementary Material

1 Supplementary Table

Table S1: Minimum inhibitory concentration (MIC) of benzohydrazides and phenylacetamide derivatives (**1-29**) against a panel of organisms

Compound	Structure	*K. p*	*P. a*	*E. c*	*S. t*	*S. e*	*S. a*	*B. s*	*M. t*
1	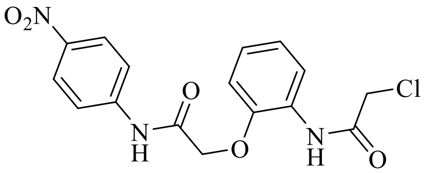	0.78	0.69	0.75	0.89	2.98	2.56	3.62	3.85
2	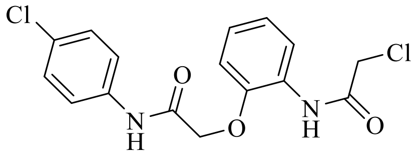	2.95	2.59	2.45	2.56	0.92	0.69	0.72	4.52
3	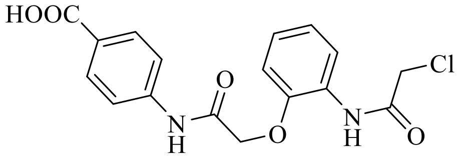	0.75	0.67	0.82	1.52	3.04	4.23	6.30	2.31
4	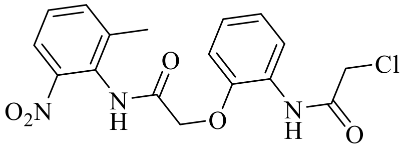	11.90	19.90	4.45	6.55	2.05	4.12	5.97	0.75
5	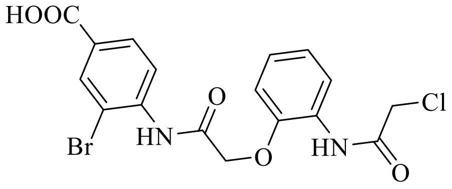	7.52	6.25	8.25	4.94	5.63	2.64	7.82	0.78
6	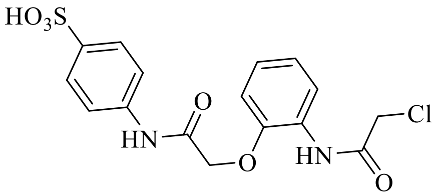	0.78	0.64	0.86	1.53	7.89	5.23	3.56	15.23
7	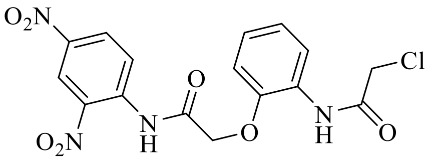	1.25	0.67	1.52	1.92	8.62	5.78	6.56	1.02

8	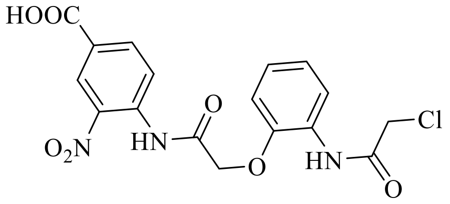	0.72	0.69	0.70	0.72	4.83	8.64	7.80	1.38
9	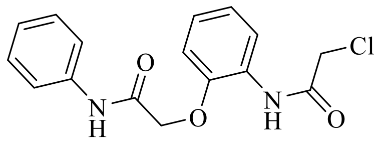	3.81	3.35	4.25	2.35	0.98	1.52	4.42	8.65
10	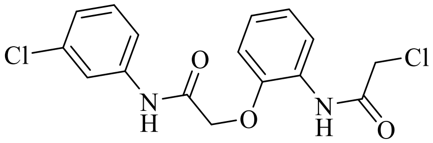	14.70	10.81	7.07	8.79	1.95	0.92	1.97	5.26
11	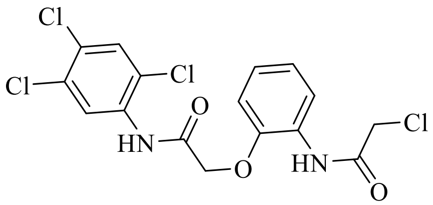	4.43	9.33	7.98	7.86	1.33	0.68	0.94	4.71
12	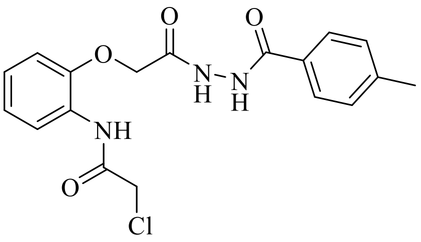	0.67	1.45	1.26	1.98	2.35	3.12	3.72	3.21
13	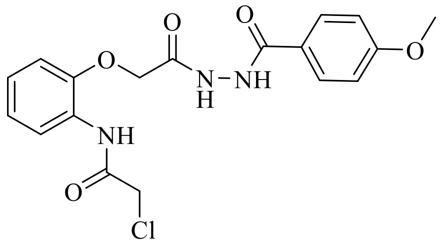	0.89	1.25	1.92	1.53	4.15	3.28	7.10	1.33
14	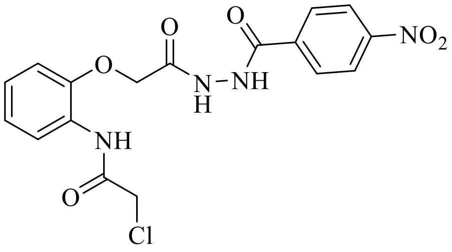	0.98	1.98	0.80	0.71	5.61	8.56	5.70	2.56
15	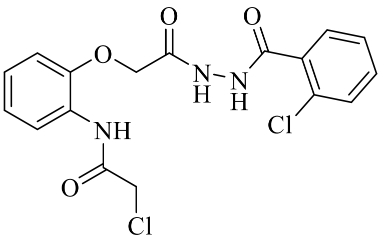	1.89	1.45	1.63	2.30	0.64	0.62	0.67	3.52

16	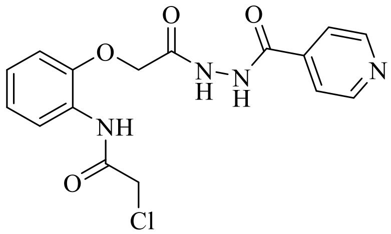	2.56	3.23	2.12	4.23	7.24	8.99	11.59	1.79
17	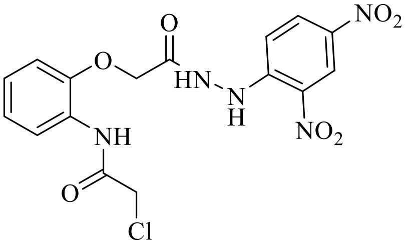	1.26	0.98	1.25	0.68	3.56	2.38	4.89	0.98
18	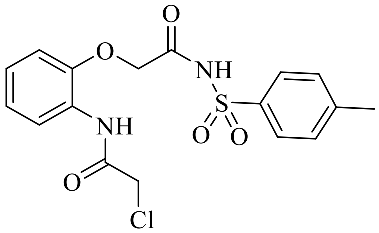	5.25	4.67	5.20	4.27	3.96	2.48	3.52	4.62
19	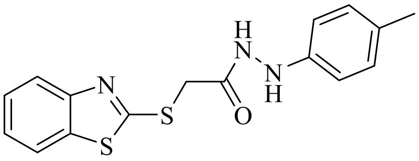	6.42	5.89	11.7	1.20	0.77	1.00	1.88	3.50
20	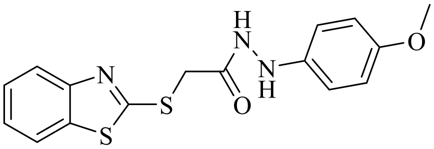	3.25	7.99	8.20	0.96	0.95	0.71	1.96	2.967
21	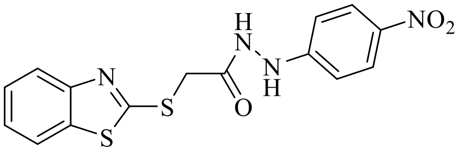	5.56	6.21	3.61	1.04	0.72	0.63	1.72	1.93
22	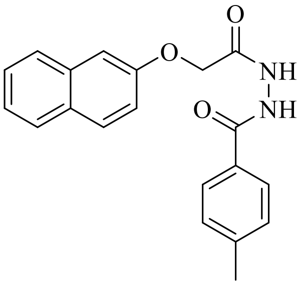	0.81	0.82	1.23	0.77	1.04	1.16	3.63	0.89
23	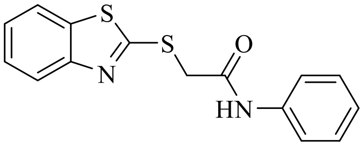	1.62	1.88	2.11	0.99	1.51	1.84	1.27	1.74
24	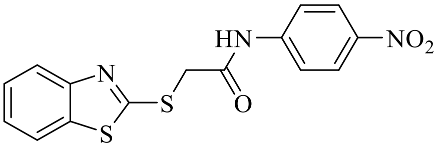	2.24	15.0	2.17	0.86	1.98	0.78	1.97	2.52

25	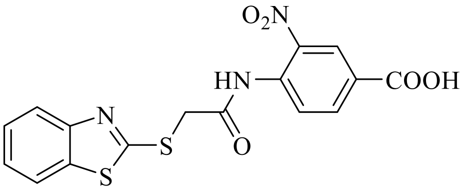	1.86	2.60	3.86	1.23	0.79	0.85	1.72	5.17
26	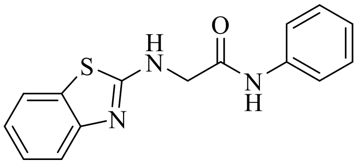	1.31	1.22	1.05	6.77	8.14	0.98	3.65	10.1
27	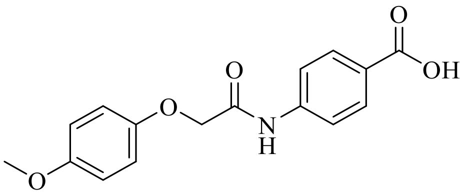	1.40	2.21	0.98	0.84	0.94	0.94	3.06	0.79
28	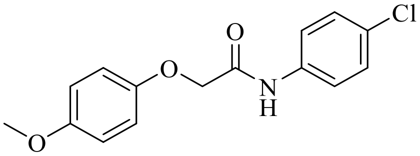	1.98	5.55	0.88	0.91	0.71	1.21	2.04	1.47
29	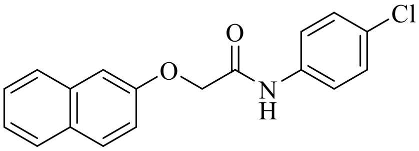	0.88	5.48	0.77	3.10	4.70	0.90	4.69	0.77
Ciprofloxacin		0.63	0.67	0.97	0.65	0.74	0.68	0.81	0.60

K. p: Klebsiella pneumonia (NCIM 2706)*; P. a: Pseudomonas aeruginosa* (NCIM 2036)*; S. t: Salmonella typhi* (NCIM 2501)*; B. s: Bacillus subtilis* (NCIM 2545)*; E. c: Escherichia coli* (NCIM 2567)*; S. e: Staphylococcus epidermis* (NCIM 2493)*; S. a: Staphylococcus aureus* (NCIM 5022)*; M. t: Mycobacterium tuberculosis* (NCIM 2984).

2 Supplementary Figures

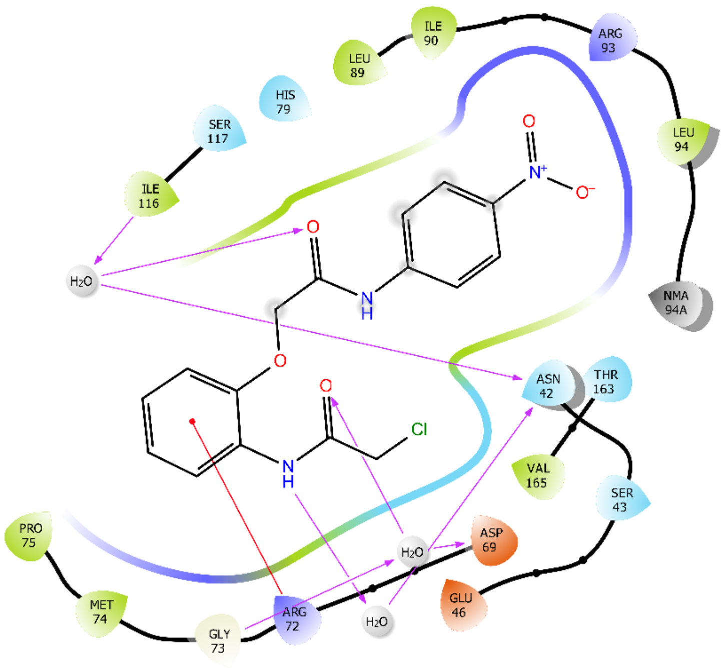

Figure S1: Two-dimensional interaction image of compound **1** against *E. coli* ParE enzyme (PDB: 3FV5)

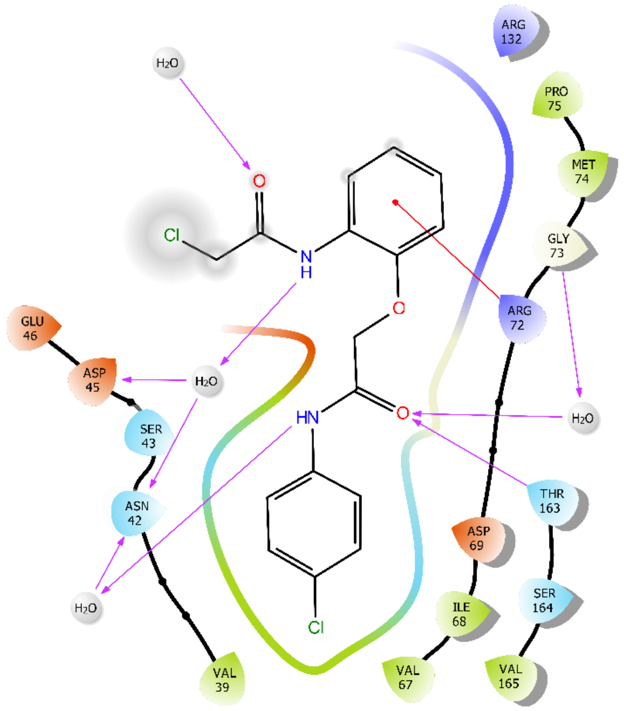

Figure S2: Two-dimensional interaction image of compound **2** against *E. coli* ParE enzyme (PDB: 3FV5)

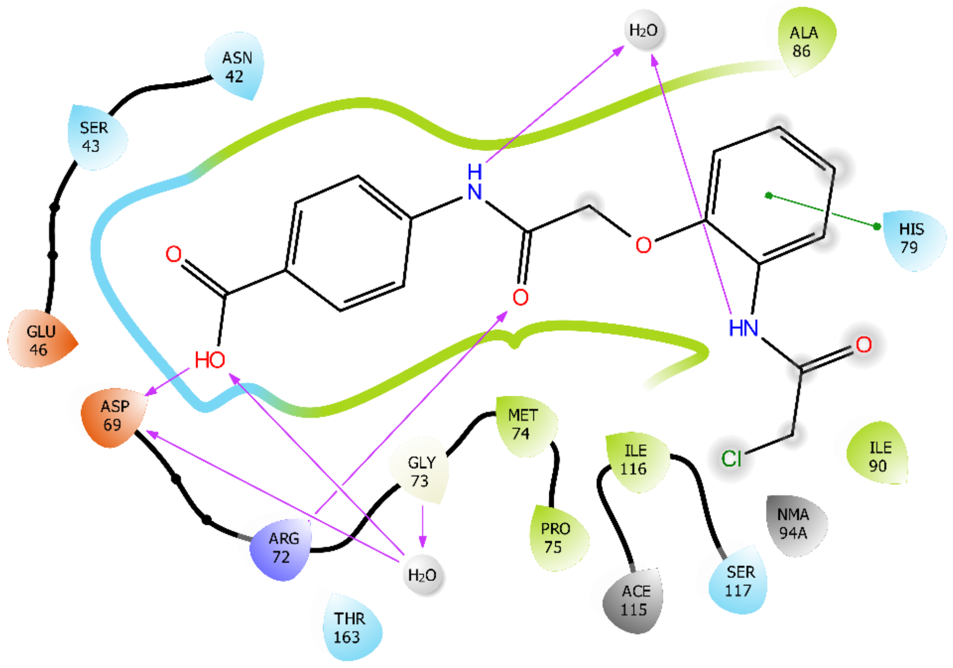

Figure S3: Two-dimensional interaction image of compound **3** against *E. coli* ParE enzyme (PDB: 3FV5)

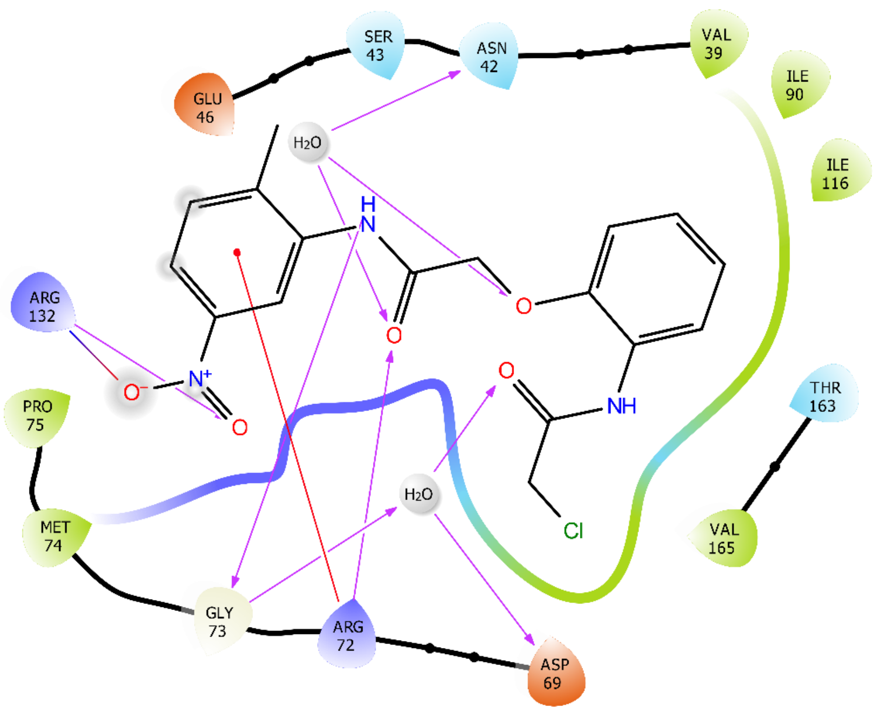

Figure S4: Two-dimensional interaction image of compound **4** against *E. coli* ParE enzyme (PDB: 3FV5)

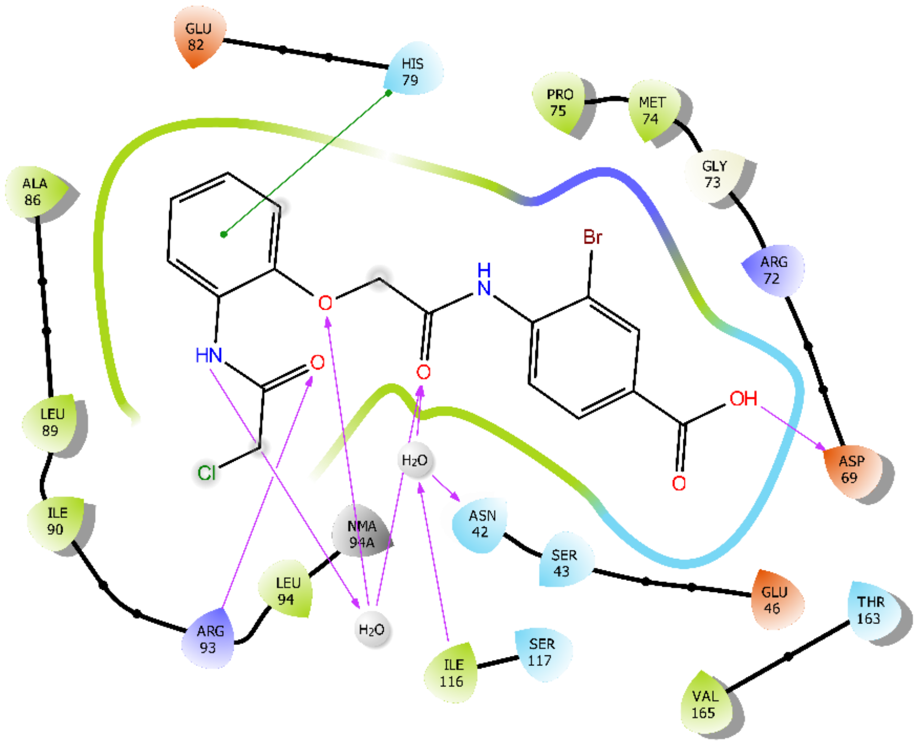

Figure S5: Two-dimensional interaction image of compound **5** against *E. coli* ParE enzyme (PDB: 3FV5)

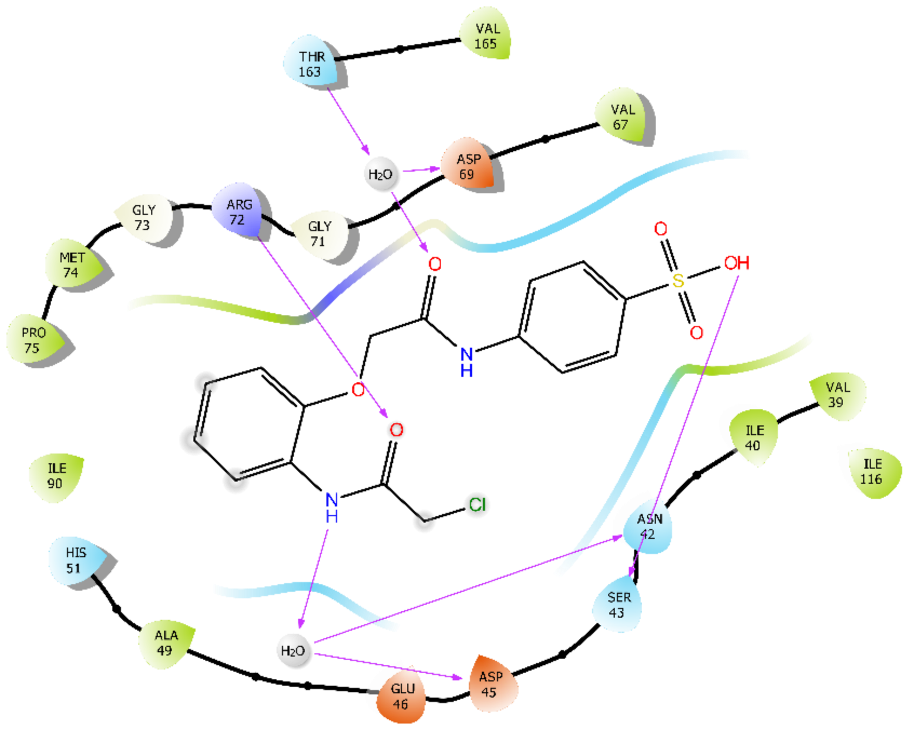

Figure S6: Two-dimensional interaction image of compound **6** against *E. coli* ParE enzyme (PDB: 3FV5)

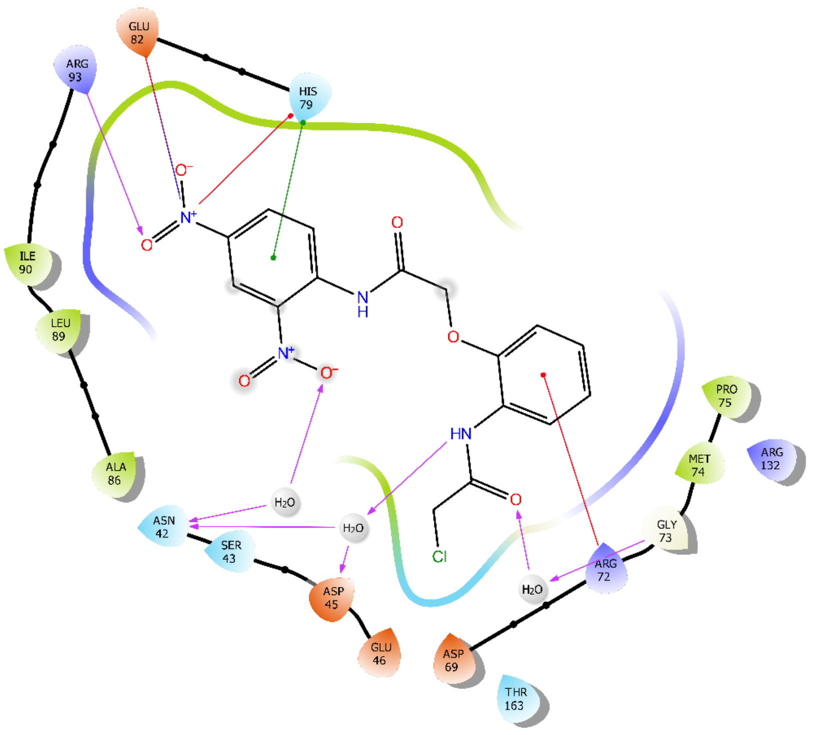

Figure S7: Two-dimensional interaction image of compound **7** against *E. coli* ParE enzyme (PDB: 3FV5)

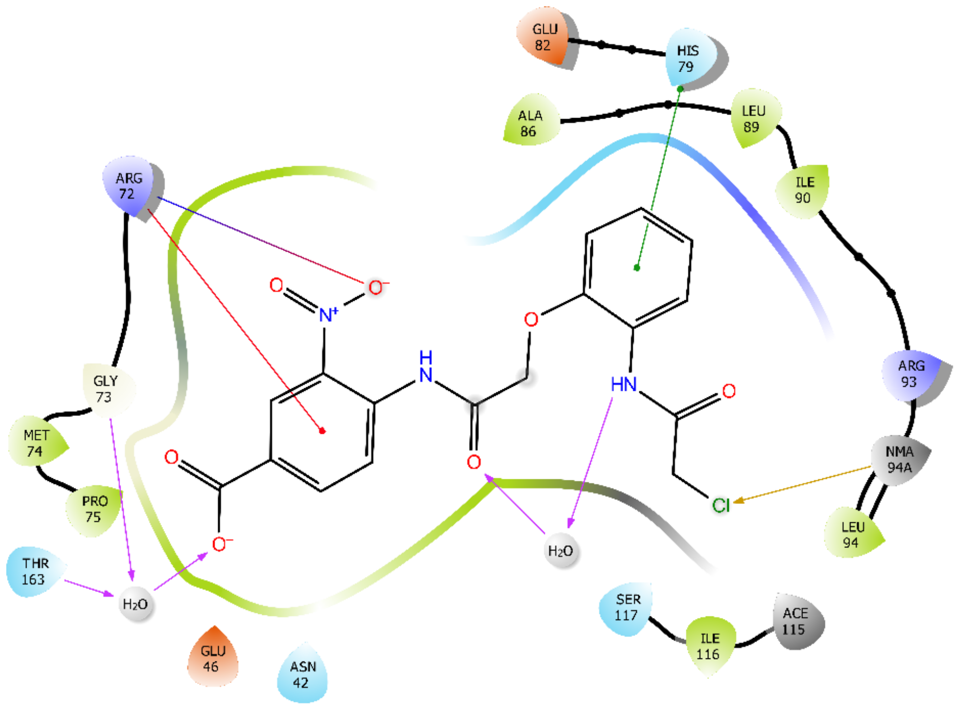

Figure S8: Two-dimensional interaction image of compound **8** against *E. coli* ParE enzyme (PDB: 3FV5)

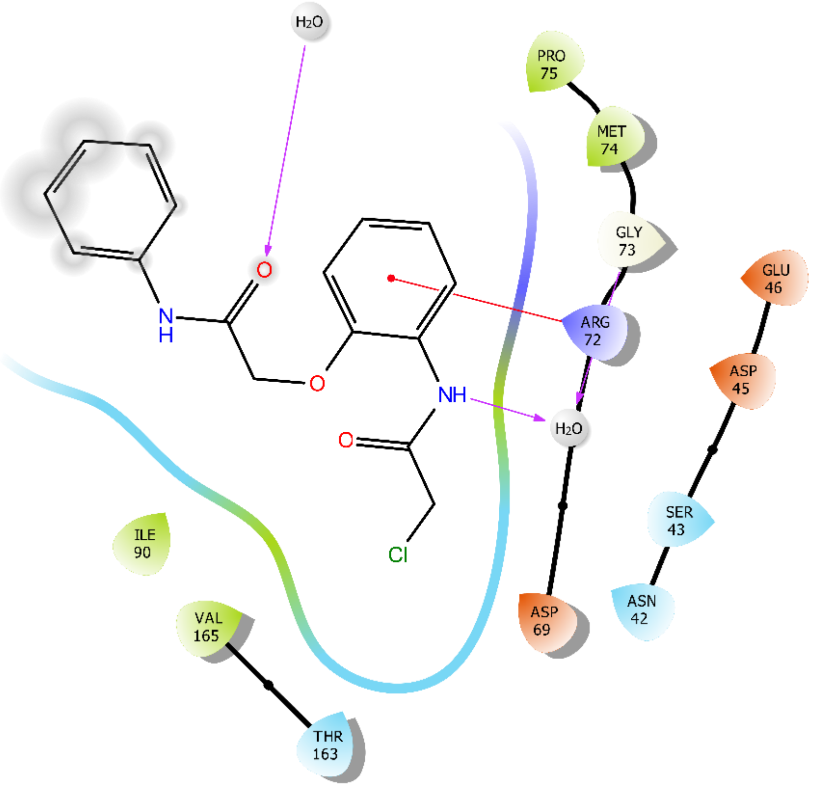

Figure S9: Two-dimensional interaction image of compound **9** against *E. coli* ParE enzyme (PDB: 3FV5)

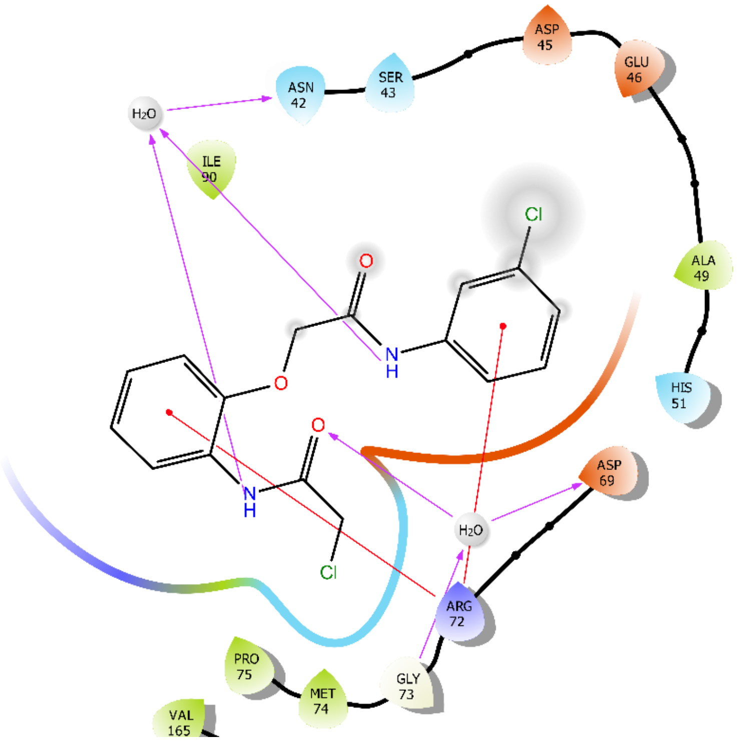

Figure S10: Two-dimensional interaction image of compound **10** against *E. coli* ParE enzyme (PDB: 3FV5)

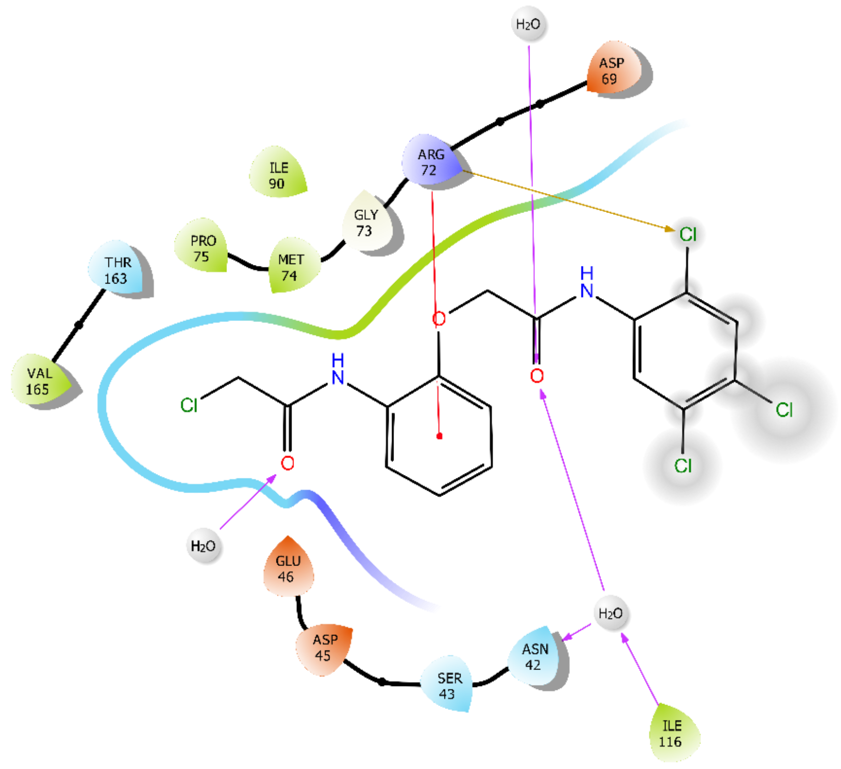

Figure S11: Two-dimensional interaction image of compound **11** against *E. coli* ParE enzyme (PDB: 3FV5)

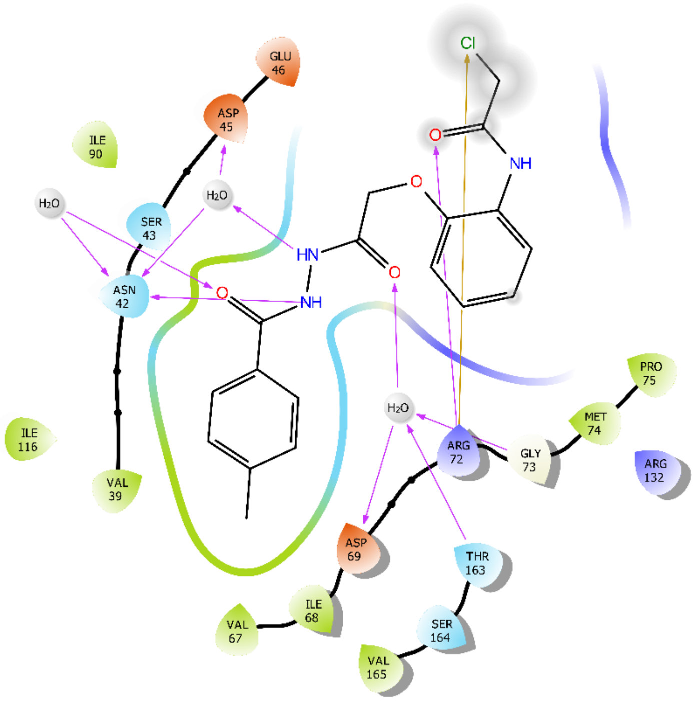

Figure S12: Two-dimensional interaction image of compound **12** against *E. coli* ParE enzyme (PDB: 3FV5)

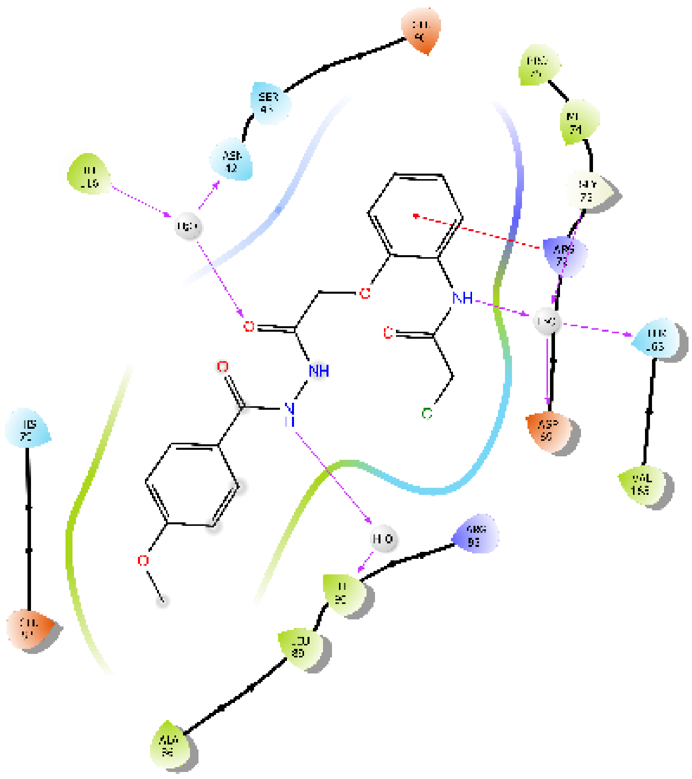

Figure S13: Two-dimensional interaction image of compound **13** against *E. coli* ParE enzyme (PDB: 3FV5)

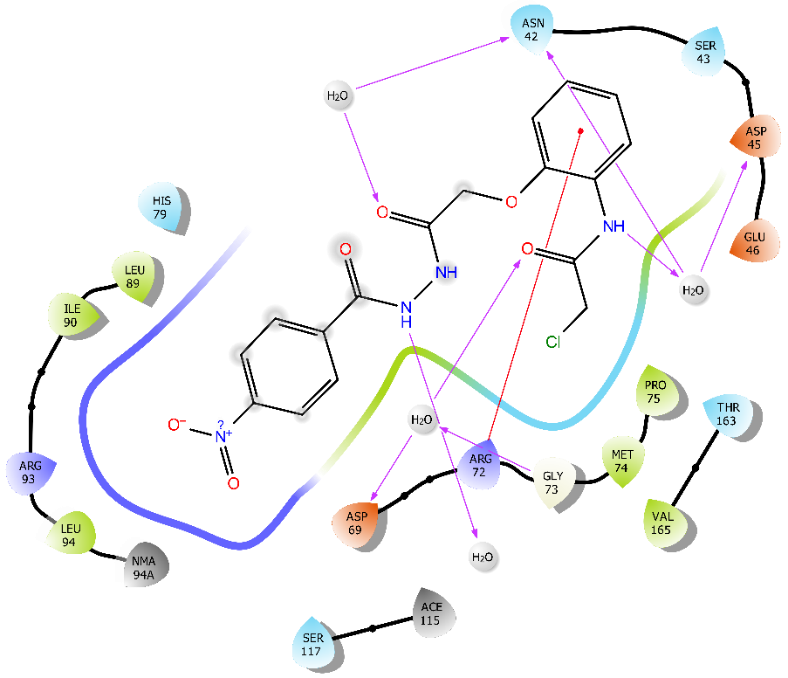

Figure S14: Two-dimensional interaction image of compound **14** against *E. coli* ParE enzyme (PDB: 3FV5)

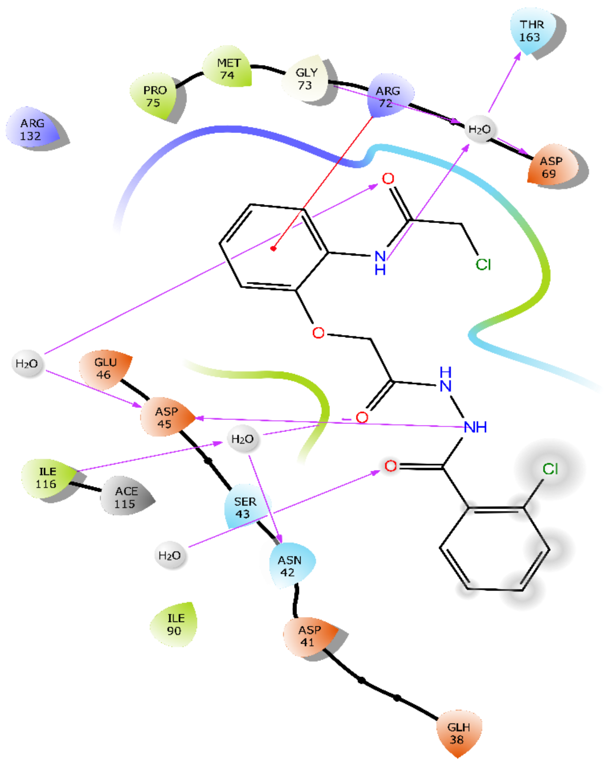

Figure S15: Two-dimensional interaction image of compound **15** against *E. coli* ParE enzyme (PDB: 3FV5)

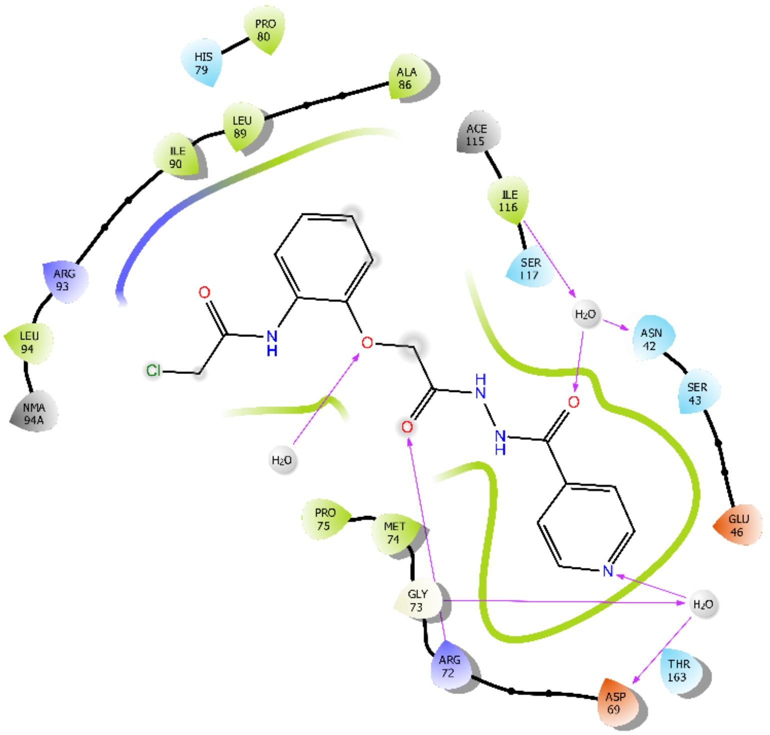

Figure S16: Two-dimensional interaction image of compound **16** against *E. coli* ParE enzyme (PDB: 3FV5)

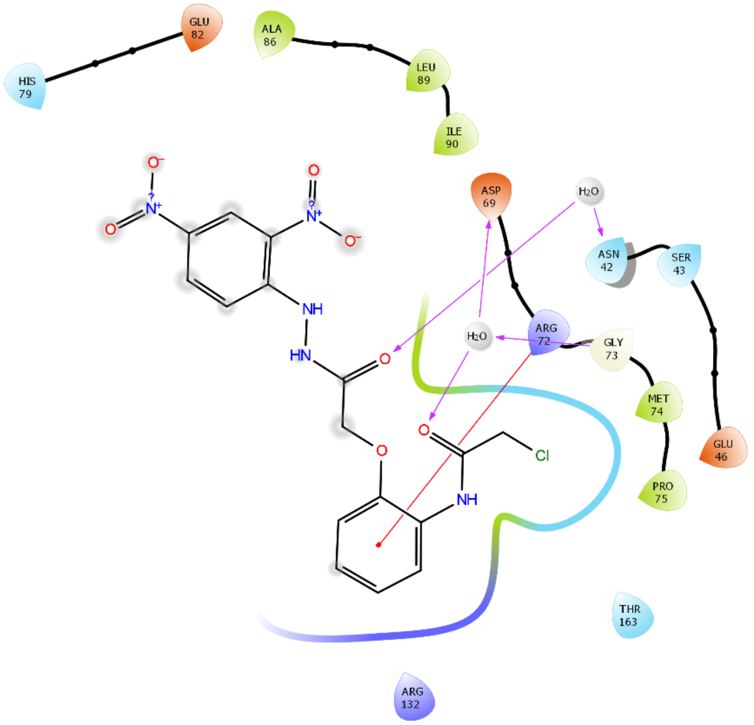

Figure S17: Two-dimensional interaction image of compound **17** against *E. coli* ParE enzyme (PDB: 3FV5)

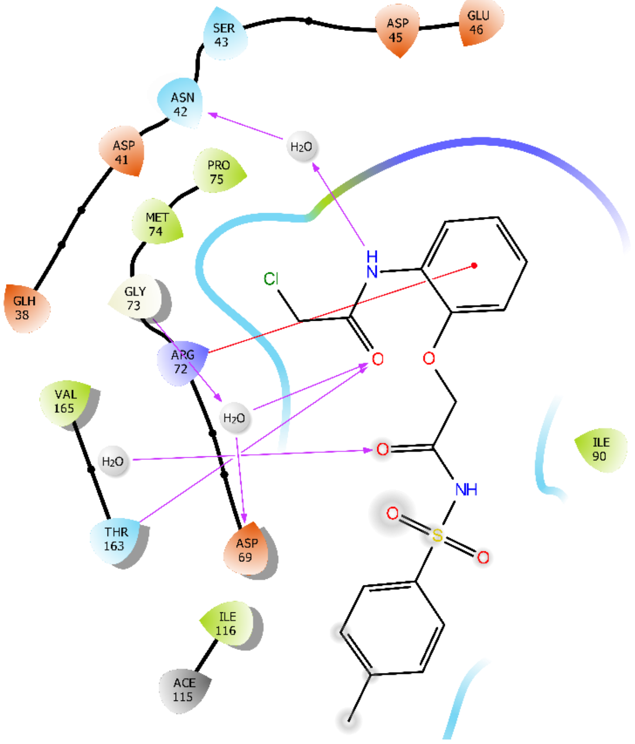

Figure S18: Two-dimensional interaction image of compound **18** against *E. coli* ParE enzyme (PDB: 3FV5)

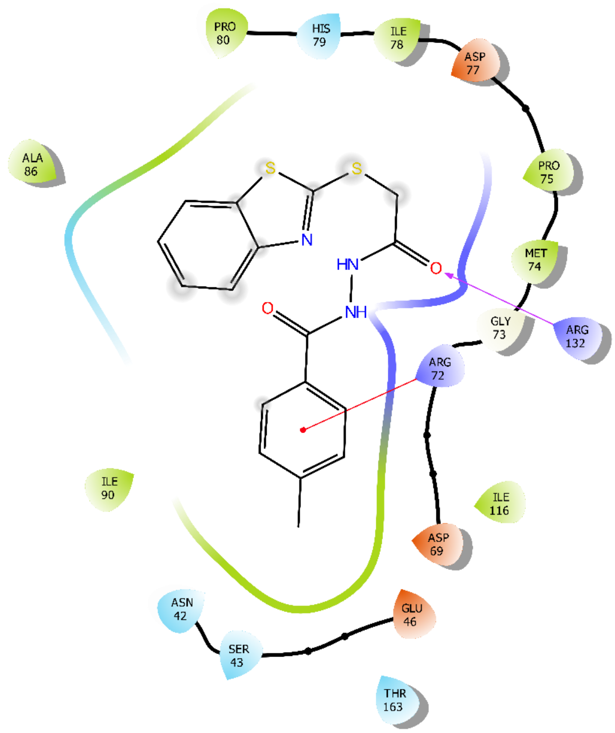

Figure S19: Two-dimensional interaction image of compound **19** against *E. coli* ParE enzyme (PDB: 3FV5)

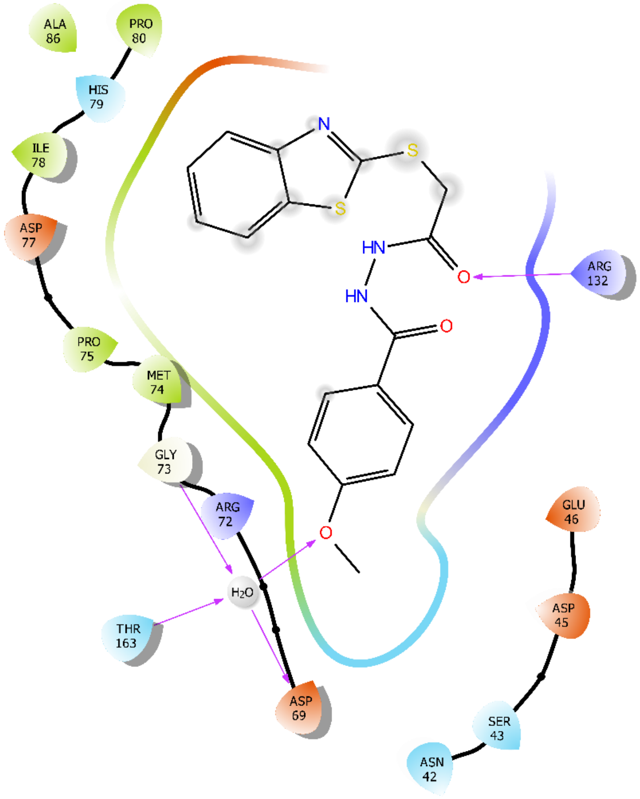

Figure S20: Two-dimensional interaction image of compound **20** against *E. coli* ParE enzyme (PDB: 3FV5)

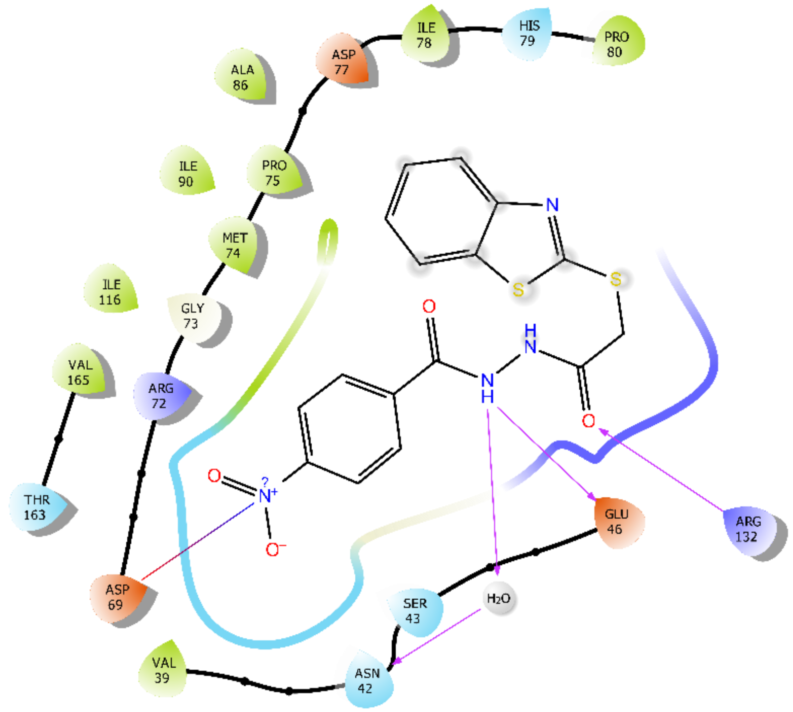

Figure S21: Two-dimensional interaction image of compound **21** against *E. coli* ParE enzyme (PDB: 3FV5)

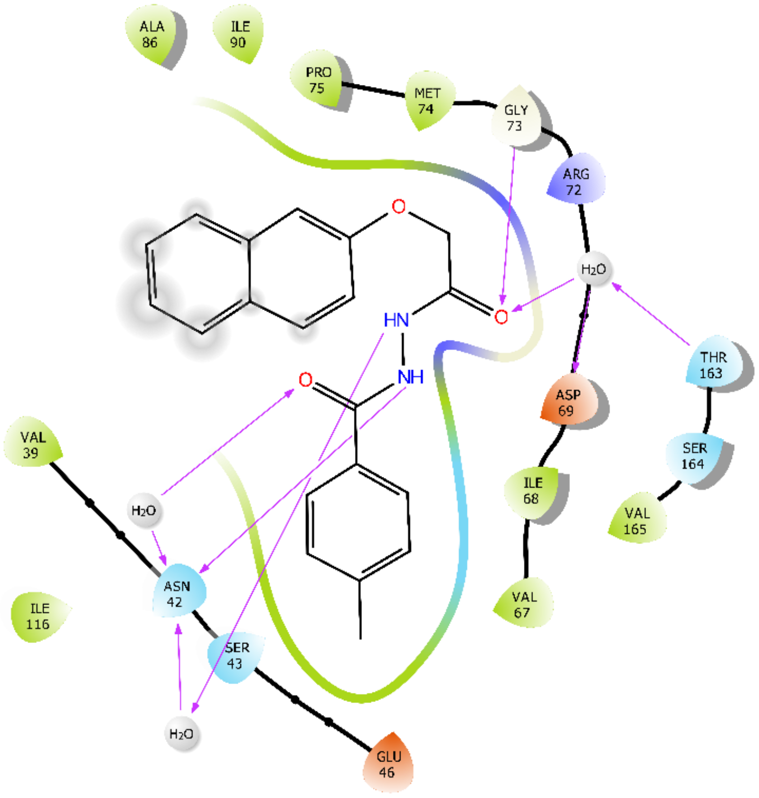

Figure S22: Two-dimensional interaction image of compound **22** against *E. coli* ParE enzyme (PDB: 3FV5)

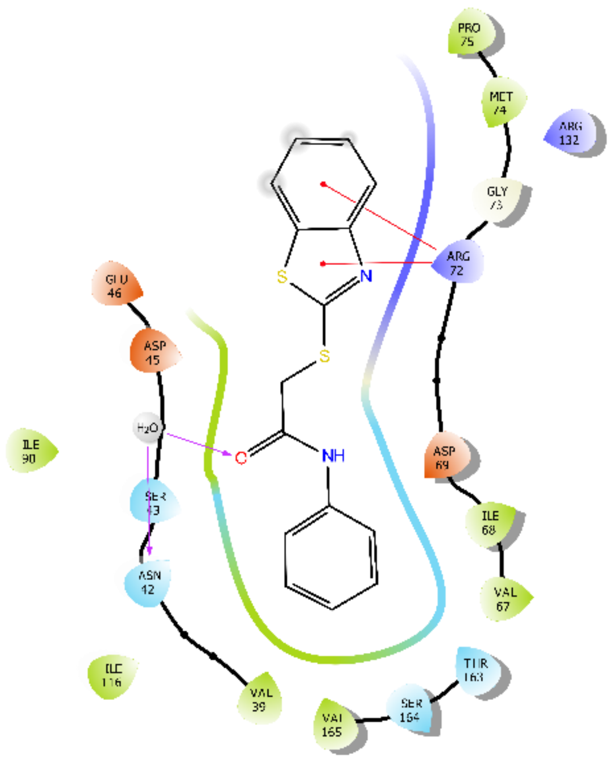

Figure S23: Two-dimensional interaction image of compound **23** against *E. coli* ParE enzyme (PDB: 3FV5)

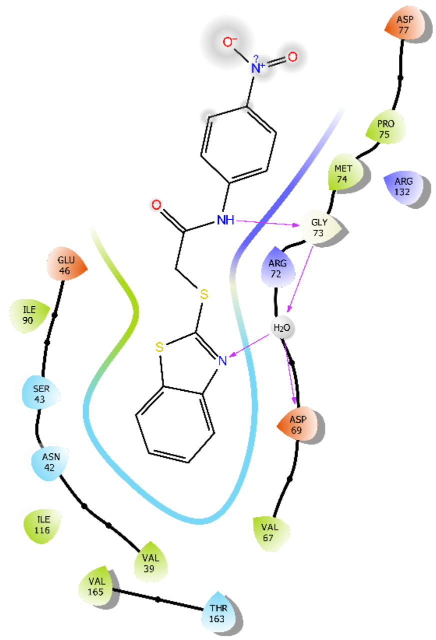

Figure S24: Two-dimensional interaction image of compound **24** against *E. coli* ParE enzyme (PDB: 3FV5)

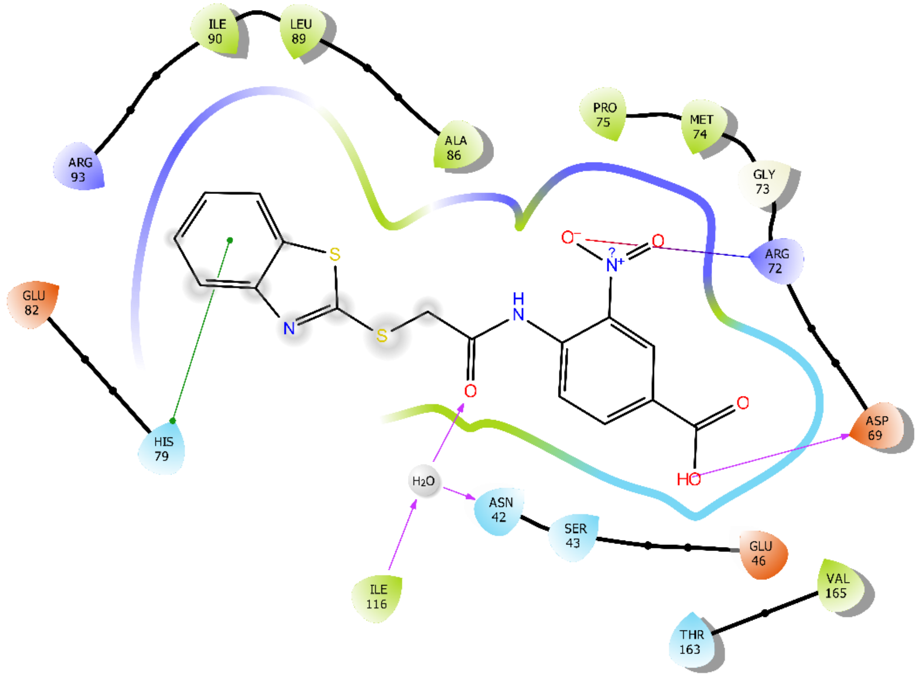

Figure S25: Two-dimensional interaction image of compound **25** against *E. coli* ParE enzyme (PDB: 3FV5)

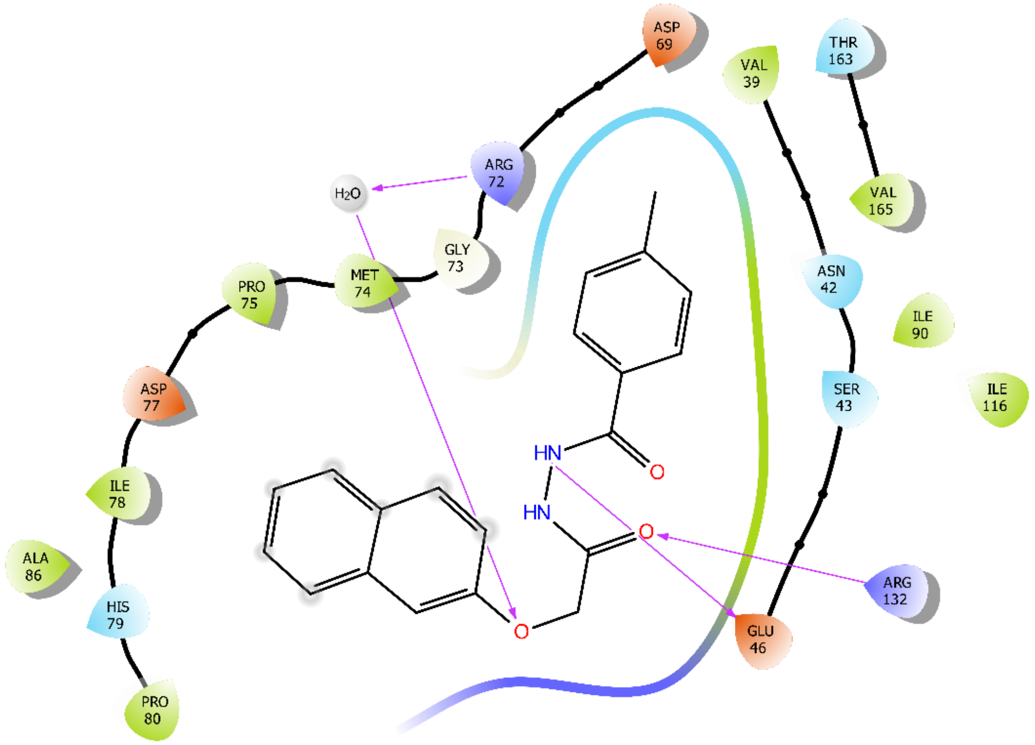

Figure S26: Two-dimensional interaction image of compound **26** against *E. coli* ParE enzyme (PDB: 3FV5)

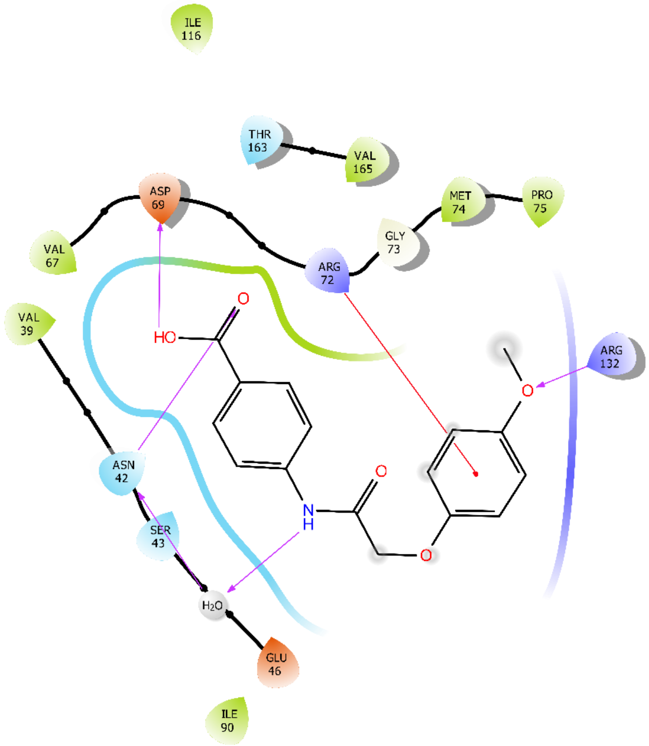

Figure S27: Two-dimensional interaction image of compound **27** against *E. coli* ParE enzyme (PDB: 3FV5)

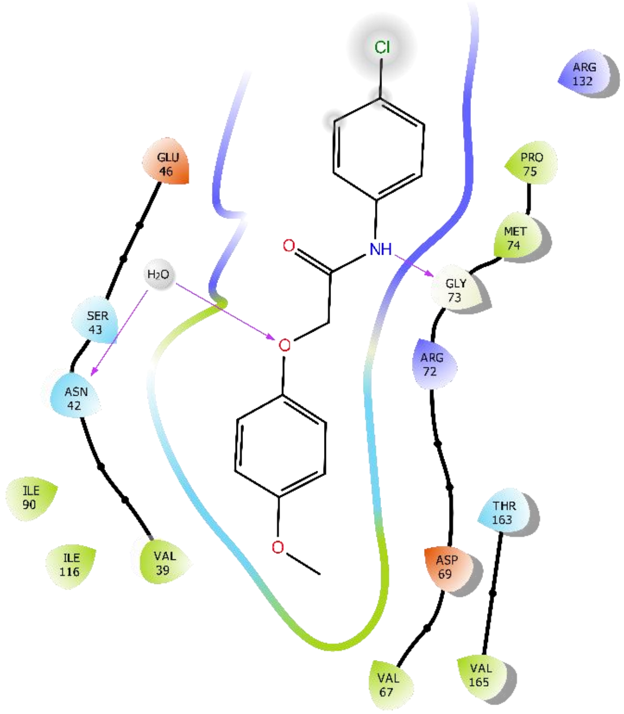

Figure S28: Two-dimensional interaction image of compound **28** against *E. coli* ParE enzyme (PDB: 3FV5)

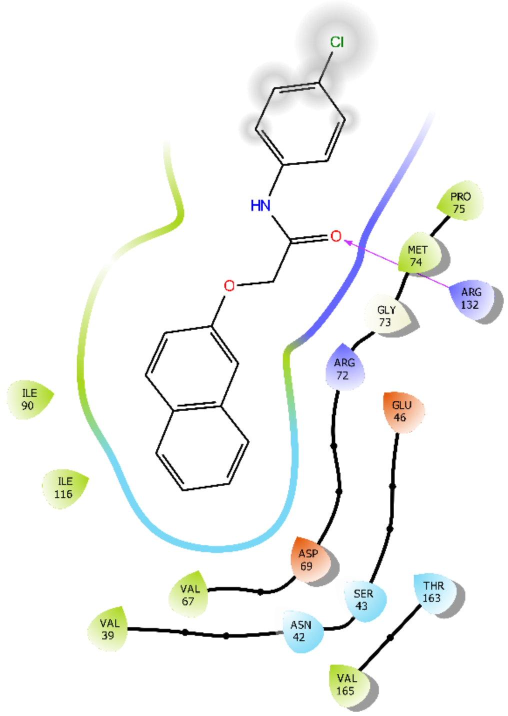

Figure S29: Two-dimensional interaction image of compound **29** against *E. coli* ParE enzyme (PDB: 3FV5)

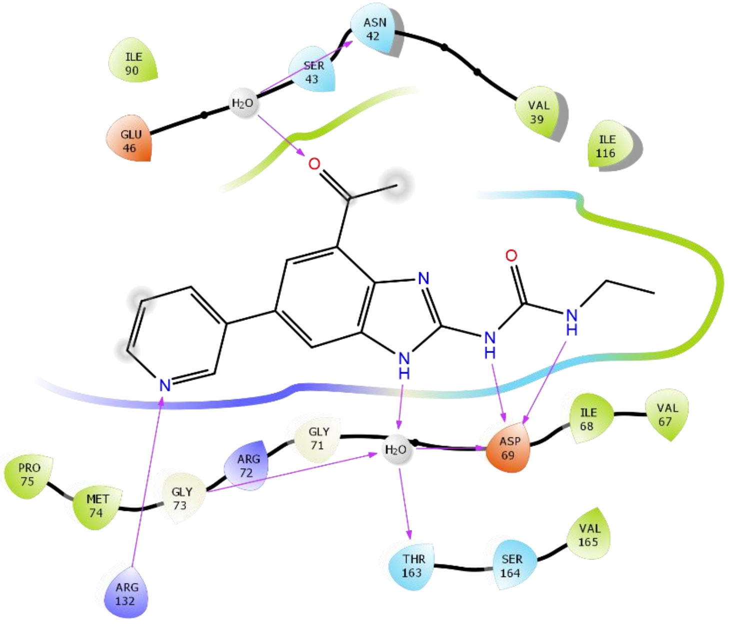

Figure S30: Two-dimensional interaction image of co-crystal against *E. coli* ParE enzyme (PDB: 3FV5)
